# Association of 13 Occupational Carcinogens in Patients With Cancer, Individually and Collectively, 1990-2017

**DOI:** 10.1001/jamanetworkopen.2020.37530

**Published:** 2021-02-18

**Authors:** Na Li, Zhen Zhai, Yi Zheng, Shuai Lin, Yujiao Deng, Grace Xiang, Jia Yao, Dong Xiang, Shuqian Wang, Pengtao Yang, Si Yang, Peng Xu, Ying Wu, Jingjing Hu, Zhijun Dai, Meng Wang

**Affiliations:** 1 Department of Oncology, The Second Affiliated Hospital of Xi’an Jiaotong University, Xi’an, China; 2 Department of Breast Surgery, The First Affiliated Hospital, College of Medicine, Zhejiang University, Hangzhou, China; 3 College of Arts and Sciences, New York University, New York; 4 Celilo Cancer Center, Oregon Health Science Center Affiliated Mid-Columbia Medical Center, The Dalles; 5 Dana-Farber Cancer Institute, Harvard Medical School, Boston, Massachusetts

## Abstract

**Importance:**

Occupational exposure to carcinogens has been shown to pose a serious disease burden at the global, regional, and national levels. Based on epidemiologic studies and clinical observations, working environment appears to have important effects on the occurrence of human malignant tumors; however, to date, no systematic articles have been published that specifically investigated cancer burden due to occupational exposure in an individual and collective manner.

**Objective:**

To estimate the degree of exposure and evaluate the cancer burden attributable to occupational carcinogens (OCs) individually and collectively by sex, age, year, and location.

**Design, Setting, and Participants:**

Cross-sectional study including data on 195 countries from the Global Burden of Diseases, Injuries, and Risk Factors Study from January 1, 1990, to December 31, 2017. Data were analyzed from June 24, 2020, to July 20, 2020.

**Exposures:**

Thirteen OCs (ie, arsenic, asbestos, benzene, beryllium, cadmium, chromium, diesel engine exhaust, formaldehyde, nickel, polycyclic aromatic hydrocarbons, silica, sulfuric acid, and trichloroethylene).

**Main Outcomes and Measures:**

The degree and change patterns of exposure as well as the attributable cancer burden, including deaths and disability-adjusted life years (DALYs), by sex, age, year, and location for 13 OCs. The calculation of the population-attributable fraction was based on past exposure in the population and relative risks.

**Results:**

Based on the GBD 2017 study, 13 OCs attributable to 7 cancer types were included. Most summary exposure values for the 13 OCs, particularly those of diesel engine exhaust (35.6% increase; 95% uncertainty interval [UI], 32.4%-38.5%) and trichloroethylene (30.3% increase; 95% UI, 27.3%-33.5%), increased from 1990 to 2017. Only exposure to asbestos decreased by 13.8% (95% UI, −26.7% to 2.2%). In 2017, 319 000 (95% UI, 256 000-382 000) cancer deaths and 6.42 million (95% UI, 5.15 million to 7.76 million) DALYs were associated with OCs combined, accounting for 61.0% (95% UI, 59.6%-62.4%) of the total cancer deaths and 48.3% (46.3% to 50.2%) of the DALYs. Among the 13 OCs, the 3 leading risk factors for cancer burden were asbestos (71.8%), silica (15.4%), and diesel engine exhaust (5.6%). For most OCs, the attributed cancer outcome was tracheal, bronchial, and lung cancer, which accounted for 89.0% of attributable cancer deaths. China (61 644 cancer deaths), the US (42 848), and Japan (20 748) accounted for the largest number of attributable cancer deaths in 2017; for DALYs, China (1.47 million), the US (0.71 million), and India (0.37 million) were the 3 leading countries.

**Conclusions and Relevance:**

Results of this study suggest that although OC exposure levels have decreased, the overall cancer burden is continuously increasing.

## Key Points

**Question:**

What is the present degree of exposure to occupational carcinogens, and are occupational carcinogens related to cancer burden over time?

**Findings:**

This cross-sectional study including data on 195 countries indicated that the exposure levels for 12 of the 13 occupational carcinogens included in this study increased from 1990 to 2017; only exposure to asbestos decreased. In 2017, all occupational carcinogens combined were associated with 319 000 cancer deaths and 6.42 million disability-adjusted life years, with asbestos, silica, and diesel engine exhaust contributing the highest levels; China, the US, and Japan accounted for the largest number of attributable cancer deaths.

**Meaning:**

Study findings suggest that increased efforts are needed in prevention of exposure to occupational carcinogens, especially further establishment of control frameworks.

## Teaser

This cross-sectional study examines the degree of occupational exposure to carcinogens globally and to the cancer-related deaths and disability-adjusted life-years associated with the exposure levels over time.

## Introduction

Occupational exposure to carcinogens has been reported to be associated with a substantial disease burden at the global, regional, and national levels, and occupational diseases are attracting increasing attention worldwide.^1^ According to a study based on the Global Burden of Diseases, Injuries, and Risk Factors Study (GBD) 2016,^2^ approximately 348 741 cancer-associated deaths in 2016 were due to occupational exposure to 14 carcinogens, including 8 cancer outcomes: larynx, nasopharynx, breast, lung, ovarian, mesothelioma, and leukemia. It has been estimated that more than 80% of cancers are associated with environmental factors, including external (chemical, physical, and biological) and internal (genetic, immune, and endocrine) sources.^3^ Among these factors, a high-risk occupational environment plays an important role in the occurrence and progression of tumors. Well-known occupational-associated tumors include asbestos-induced lung cancer^4^ and mesothelioma,^5^ benzene-induced bladder cancer,^6^ and petroleum pitch–induced skin cancer.^7^ Lung cancer accounts for the largest proportion of occupational-associated cancers.^2^

The series of GBD studies conducted by the Institute of Health Metrics and Evaluation started in 2010 with a focus on risk outcome.^8^ The study summarized the outcomes of occupational carcinogens (OCs), and the work has since been updated several times at national and global levels.^1,9^ However, to our knowledge, no systematic studies have been published that specifically investigate cancer burden associated with occupational exposure in an individual and collective manner.

In this study, we used data from GBD 2017 to estimate the degree of exposure to OCs and evaluate the specific cancer burden attributable to OCs individually and collectively by sex, age, and location. We analyzed the association between attributable burden and social development from January 1, 1990, to December 31, 2017, present the global and regional estimates of 13 OCs, and report cancer burdens attributable to each OC in terms of deaths and disability-adjusted life years (DALYs).

## Methods

### GBD Overview

The Global Health Data Exchange query tool is a continuing global collaboration that uses epidemiologic data to provide a comparative assessment of health loss from 328 diseases across 195 countries and territories within 21 regions.^10^ Data on the OC-attributable cancer burden were obtained from the Global Health Data Exchange. The GBD study organized environmental, occupational, behavioral, and metabolic risk factors into 5 hierarchical levels through a comparative risk assessment method (eTable 1 in the Supplement).

### Study Data and Participants

Based on the GBD 2017 study, 13 work-environment carcinogens attributable to 7 cancer types were included in our cross-sectional study: tracheal, bronchial, and lung cancer (exposure to arsenic, asbestos, beryllium, cadmium, chromium, diesel engine exhaust, nickel, polycyclic aromatic hydrocarbons, and silica), leukemia (exposure to benzene and formaldehyde), larynx cancer (exposure to asbestos and sulfuric acid), mesothelioma (exposure to asbestos), nasopharynx cancer (exposure to formaldehyde), ovarian cancer (exposure to asbestos), and kidney cancer (exposure to trichloroethylene). Each combination of risk and outcome included in the GBD is recognized as a risk-outcome pair, which was obtained on the basis of evidence rules (eTable 2 in the Supplement). Data on OC-attributable cancer deaths and DALYs were obtained, along with their respective age-standardized rates, and the summary exposure value of each OC from January 1, 1990, to December 31, 2017. Data were analyzed from June 24, 2020, to July 20, 2020.

This study was based on the GBD database and does not contain identifiable personal information. Therefore, a waiver of informed consent was reviewed and approved by the University of Washington institutional review board. Both sexes were included, and race/ethnicity was not reported. This study followed the Strengthening the Reporting of Observational Studies in Epidemiology (STROBE) reporting guideline for cross-sectional studies.

### General Estimation 

The general methods used in this research have been described in detail elsewhere.^11^ Herein, we briefly summarize the methods and provide more detailed information on OC analysis. Summary exposure value is a measure of a population’s risk-weighted exposure or risk-weighted prevalence of a single exposure and was used to estimate the exposure value of the OCs involved. Summary exposure values range from 0% to 100%: a value of 0% indicates no exposure and 100% indicates maximum possible risk for the entire population. An increase in the summary exposure value indicates an increase in exposure to a given risk factor, whereas a decrease indicates a decrease in exposure. The social demographic index (SDI) is an indicator of social development status based on total fertility rate, average years of education for individuals aged 15 years or older, and the lagging income distribution per capita. Social demographic index scores range from 0 (highest fertility, lowest income, and lowest educational level) to 1 (highest educational level and lowest fertility). Each GBD location has an annual SDI score, and countries are divided into 5 SDI quintiles (high, high-medium, medium, medium-low, and low levels). In this study, the association between the SDI value and OC-attributable cancer burden was investigated.

### Attributable Burden Estimation

The relative risks used in the analyses were selected from the GBD database for the burden estimation. For each risk-outcome pair, the same relative risk estimates were used for both sexes and for all age groups (eTable 3 in the Supplement). Attributable DALYs were estimated as the total resulting DALY multiplied by the population-attributable fraction (PAF) for the risk-outcome pair for each age, sex, cause, and location. The same logic was applied to estimating attributable deaths, years of life lost, and years lived with disability. The PAF for each separate risk-outcome pair was estimated independently along with the combined burdens of all types of cancer attributable to the specific risk factor, whether directly or indirectly (eTable 4 in the Supplement). The PAFs for each age-sex-country group of OCs were calculated based on the formula estimates in Levin’s^12^ study:
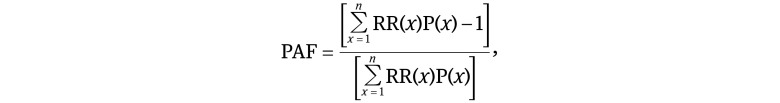
where RR(*x*) represents the relative risk corresponding to level *x* exposure and P(*x*) represents the proportion of persons at level *x* exposure in the relevant population.

### Evidence Rules

Evidence rules means that the association of the risk-outcome pair has been verified by published meta-analyses or pooled studies; if these did not exist, key single studies were also adopted. If single studies were used, the chosen study was the best-quality study with exposure circumstances that were assessed as most closely matching those assumed in the GBD study.

### Statistical Analysis

Based on the GBD 2017 study, 13 OCs attributable to 7 cancer types were included. Data on annual OC-attributable cancer deaths and DALYs were obtained, along with their respective age-standardized rates, and the summary exposure value of each OC from 1990 to 2017. According to the SDI, countries are divided into 5 quintiles (high, high-medium, medium, medium-low, and low levels). In this study, the association between the SDI value and OC-attributable cancer burden was investigated with Pearson correlation analysis. A 2-sided P<.05 was considered statistically significant. All statistical analyses were performed using the R program, version 4.0.2 (R Foundation for Statistical Computing).

## Results

### Global Exposure to OCs

Table 1 provides the age-standardized summary exposure values for 13 OCs at a global level based on sex for 1990, 2007, and 2017. From 1990 to 2017, summary exposure values for 2 OCs increased by more than 30% among both men and women: exposure to diesel engine exhaust increased by 35.6% (95% uncertainty interval [UI], 32.4%-38.5%) and exposure to trichloroethylene increased by 30.3% (95% UI, 27.3%-33.5%). Summary exposure values for 4 OCs increased by more than 20%: polycyclic aromatic hydrocarbons (27.7%; 95% UI, 23.9%-31.4%), chromium (27.3%; 95% UI, 23.5%-31.3%), benzene (26.0%; 95% UI, 19.0%-41.9%), and formaldehyde (21.5%; 95% UI, 17.5%-25.6%). Among the 13 OCs, only exposure to asbestos decreased among both sexes by 13.8% (95% UI, −26.7% to 2.2%). Summary exposure values for exposure to nickel increased by 3.1% (95% UI, −3.5% to 10.2%) for men and decreased by 0.2% (95% UI, −7.7% to 20.7%) for women, and summary exposure values for exposure to silica increased by 9.0% (95% UI, 3.5%-21.2%) for men and decreased by 7.4% (−12.4% to 2.2%) for women.

**Table 1.** Global Age-Standardized Summary Exposure Value for OCs in Patients With Cancer^a^

OC exposure	Men									Women								
	% (95% UI)				% Change (95% UI)				% (95% UI)					% Change (95% UI)			
	1990	2007	2017	1990-2007		2007-2017	1990-2017	1990			2007	2017	1990-2007		2007-2017	1990-2017
Arsenic	0.32 (0.09 to 0.61)	0.33 (0.10 to 0.60)	0.34 (0.10 to 0.61)	2.1 (−1.6 to 13.8)		3.0 (0.3 to 8.7)	5.2 (−1.6 to 22.8)	0.29 (0.08 to 0.57)			0.30 (0.09 to 0.57)	0.31 (0.10 to 0.57)	3.1 (−1.8 to 18.4)		2.1 (−1.9 to 9.2)	5.2 (−1.9 to 27.8)
Asbestos	2.67 (2.33 to 3.05)	2.47 (2.38 to 2.60)	2.36 (2.28 to 2.45)	−7.7 (−15.8 to 3.7)		−4.2 (−8.9 to −0.3)	−11.6 (−23.0 to 2.5)	0.98 (0.76 to 1.25)			0.79 (0.73 to 0.88)	0.74 (0.70 to 0.79)	−18.6 (−31.0 to −2.3)		−6.6 (−13.5 to −0.8)	−24.9 (−40.0 to −3.8)
Benzene	0.54 (0.26 to 1.11)	0.60 (0.31 to 1.20)	0.65 (0.35 to 1.27)	11.1 (7.2 to 19.4)		7.9 (5.7 to 11.9)	19.8 (13.8 to 33.1)	0.51 (0.22 to 1.10)			0.60 (0.28 to 1.26)	0.68 (0.34 to 1.37)	18.2 (13.6 to 28.2)		12.4 (8.9 to 18.8)	32.8 (24.1 to 51.9)
Beryllium	0.06 (0.06 to 0.06)	0.07 (0.07 to 0.07)	0.07 (0.07 to 0.07)	8.5 (6.8 to 10.3)		4.8 (3.5 to 6.1)	13.8 (11.3 to 16.1)	0.05 (0.05 to 0.06)			0.06 (0.06 to 0.06)	0.07 (0.06 to 0.07)	13.4 (10.8 to 16.0)		6.4 (4.6 to 8.2)	20.6 (17.0 to 24.0)
Cadmium	0.13 (0.12 to 0.13)	0.14 (0.14 to 0.14)	0.15 (0.14 to 0.15)	10.9 (7.8 to 14.2)		7.0 (4.2 to 9.5)	18.6 (14.6 to 23.0)	0.11 (0.11 to 0.11)			0.13 (0.12 to 0.13)	0.14 (0.13 to 0.14)	13.7 (8.9 to 18.8)		7.0 (2.9 to 11.7)	21.7 (15.3 to 28.3)
Chromium	0.27 (0.26 to 0.27)	0.31 (0.30 to 0.31)	0.34 (0.33 to 0.35)	14.8 (11.6 to 18.2)		9.6 (6.9 to 12.1)	25.7 (21.6 to 30.3)	0.23 (0.23 to 0.24)			0.28 (0.26 to 0.29)	0.30 (0.29 to 0.32)	17.5 (12.3 to 22.8)		10.0 (5.7 to 15.0)	29.2 (22.2 to 36.4)
Diesel engine exhaust	1.51 (1.48 to 1.54)	1.83 (1.80 to 1.87)	2.07 (2.03 to 2.11)	21.6 (18.8 to 24.5)		13.0 (10.7 to 15.2)	37.4 (33.5 to 41.5)	0.94 (0.92 to 0.97)			1.12 (1.09 to 1.15)	1.25 (1.22 to 1.29)	19.1 (15.6 to 22.6)		11.9 (8.8 to 15.0)	33.3 (28.2 to 38.4)
Formaldehyde	0.59 (0.59 to 0.60)	0.66 (0.63 to 0.68)	0.71 (0.68 to 0.74)	12.4 (9.0 to 16.1)		8.1 (5.1 to 10.9)	21.5 (17.0 to 26.2)	0.49 (0.47 to 0.51)			0.55 (0.53 to 0.58)	0.60 (0.57 to 0.63)	12.8 (7.2 to 18.5)		8.0 (3.5 to 13.0)	21.8 (15.0 to 28.8)
Nickel	0.35 (0.08 to 1.10)	0.35 (0.09 to 1.08)	0.36 (0.10 to 1.08)	0.8 (−3.9 to 11.9)		2.4 (−0.4 to 8.2)	3.1 (−3.5 to 20.2)	0.28 (0.06 to 0.90)			0.28 (0.07 to 0.85)	0.28 (0.07 to 0.84)	−0.3 (−5.5 to 14.2)		0.2 (−3.5 to 8.5)	−0.2 (−7.7 to 20.7)
PAHs	0.55 (0.54 to 0.56)	0.64 (0.62 to 0.65)	0.70 (0.68 to 0.72)	14.8 (11.9 to 18.0)		9.7 (7.2 to 12.0)	26.0 (22.0 to 30.4)	0.48 (0.47 to 0.49)			0.56 (0.54 to 0.59)	0.62 (0.59 to 0.65)	17.5 (12.5 to 22.5)		10.5 (6.2 to 15.1)	29.8 (23.2 to 36.3)
Silica	3.71 (1.52 to 9.28)	3.86 (1.70 to 9.29)	4.05 (1.85 to 9.56)	4.0 (0.3 to 11.5)		4.8 (2.2 to 8.8)	9.0 (3.5 to 21.2)	2.50 (1.00 to 6.31)			2.36 (1.01 to 5.64)	2.32 (1.01 to 5.48)	−5.4 (−9.1 to 2.2)		−2.1 (−5.2 to 2.8)	−7.4 (−12.4 to 2.2)
Sulfuric acid	0.65 (0.39 to 1.34)	0.68 (0.42 to 1.36)	0.69 (0.44 to 1.35)	4.2 (0.1 to 10.2)		2.3 (−0.4 to 5.6)	6.5 (0.2 to 15.1)	0.58 (0.34 to 1.20)			0.61 (0.38 to 1.23)	0.61 (0.39 to 1.22)	5.3 (0.2 to 12.1)		1.2 (−2.5 to 6.1)	6.5 (−0.6 to 16.4)
Trichloroethylene	0.16 (0.15 to 0.16)	0.18 (0.18 to 0.19)	0.20 (0.20 to 0.21)	16.8 (12.3 to 19.7)		10.2 (8.2 to 12.2)	28.7 (25.2 to 32.4)	0.13 (0.13 to 0.13)			0.16 (0.15 to 0.16)	0.17 (0.17 to 0.18)	19.6 (15.6 to 23.8)		10.7 (7.4 to 14.6)	32.4 (26.8 to 38.1)

Abbreviations: OC, occupational carcinogen; PAHs, polycyclic aromatic hydrocarbons; UI, uncertainty interval.

^a^ Risks are reported in order of percentage change for both sexes combined, 1990-2017.

### Geographic-Attributable Burden for OCs

Cancer deaths and DALYs could be partially attributed to all OCs combined. Globally, 61.0% (95% UI, 59.6%-62.4%) of cancer deaths and 48.3% (95% UI, 46.3%-50.2%) of DALYs could be attributed to OCs in 2017. Occupational carcinogen–attributable age-standardized cancer deaths decreased by 2.7% (95% UI, −5.7% to 0.1%) and age-standardized DALY rates decreased by 2.8% (95% UI, −6.0% to 0.0%) between 2007 and 2017. Table 2 presents the number of OC-attributable cancer deaths and DALYs for each related cancer type. Among the 13 OCs, the 3 leading risk factors for cancer burden were asbestos (71.8%), silica (15.4%), and diesel engine exhaust (5.6%). For most OCs, the attributed cancer outcome was tracheal, bronchial, and lung cancer, which accounted for 89.0% of attributable cancer deaths.

**Table 2.** Global All-Age Deaths and DALYs of Patients With Cancer Attributable to Each Occupational Carcinogen and Outcome for Both Sexes, 2007-2017

Occupational carcinogen	Deaths, No. × 10^3^ (95% UI)							DALYs		
	2007	2017	Change, 2007-2017, % (95% CI)	Age-standardized change, 2007-2017, % (95% CI)	2007	2017	Change, 2007-2017, % (95% CI)		Age-standardized change, 2007-2017, % (95% CI)
Total neoplasms	258 (207 to 309)	319 (256 to 382)	23.7 (20.5 to 33.7)	−2.7 (−5.7 to 0.1)	5310 (4270 to 6421)	6423 (5151 to 7761)	21.0 (16.4 to 25.0)		−2.8 (−6.0 to −0.0)
Exposure to arsenic									
Neoplasms	7 (1 to 13)	9 (2 to 16)	33.8 (27.3 to 55.2)	6.6 (1.9 to 24.2)	189 (43 to 346)	192 (65 to 436)	29.5 (23.2 to 49.0)		4.8 (0.0 to 21.3)
Tracheal, bronchus, and lung cancer	7 (1 to 13)	9 (2 to 16)	33.8 (27.3 to 55.2)	3.9 (−0.4 to 21.1)	189 (43 to 346)	192 (65 to 436)	29.5 (23.2 to 49.0)		3.6 (−0.7 to 20.1)
Exposure to benzene									
Neoplasms	2 (1 to 3)	2 (1 to 3)	18.6 (13.0 to 24.6)	5.0 (−0.8 to 11.0)	73 (23 to 120)	84 (26 to 137)	15.4 (10.1 to 21.2)		5.2 (−0.2 to 10.8)
Leukemia	2 (1 to 3)	2 (1 to 3)	18.6 (13.0 to 24.6)	11.0 (5.3 to 16.7)	73 (23 to 120)	84 (26 to 137)	15.4 (10.1 to 21.2)		13.0 (6.6 to 20.8)
Exposure to asbestos									
Neoplasms	192 (145 to 240)	229 (173 to 285)	19.5 (14.5 to 23.7)	−6.4 (−9.9 to −3.4)	3352 (2504 to 4246)	3863 (2913 to 4873)	15.2 (9.7 to 19.9)		−8.0 (−12.0 to −4.8)
Larynx cancer	3 (2 to 5)	4 (2 to 6)	20.9 (13.4 to 27.0)	−2.0 (−6.9 to 2.3)	63 (35 to 95)	74 (41 to 112)	17.1 (9.4 to 23.1)		−3.1 (−8.2 to 1.4)
Mesothelioma	21 (21 to 23)	27 (27 to 28)	27.7 (20.5 to 33.7)	−0.2 (−0.5 to 0.0)	464 (434 to 512)	569 (542 to 598)	22.4 (14.5 to 29.3)		−0.6 (−1.2 to −0.2)
Ovarian cancer	5 (3 to 8)	6 (3 to 10)	17.4 (9.6 to 24.2)	−13.1 (−18.5 to −8.9)	88 (44 to 138)	100 (49 to 156)	14.3 (6.4 to 21.4)		−15.3 (−21.0 to −10.9)
Tracheal, bronchus, and lung cancer	161 (115 to 268)	191 (137 to 247)	18.5 (13.5 to 22.6)	−9.6 (−12.9 to −6.6)	2737 (1906 to 3588)	3120 (2176 to 4106)	14.0 (8.7 to 18.4)		−10.3 (−13.9 to −7.0)
Exposure to beryllium									
Neoplasms	0 (0 to 0)	0 (0 to 0)	45.4 (38.9 to 52.2)	16.2 (12.0-20.4)	5 (5 to 6)	8 (6 to 9)	40.1 (33.4 to 47.3)		13.9 (9.4 to 18.3)
Tracheal, bronchus, and lung cancer	0 (0 to 0)	0 (0 to 0)	45.4 (38.9 to 52.2)	13.3 (10.2 to 16.6)	5 (5 to 6)	8 (6 to 9)	40.1 (33.4 to 47.3)		12.5 (9.3 to 16.0)
Exposure to cadmium									
Neoplasms	0 (0 to 1)	1 (1 to 1)	47.7 (40.1 to 56.0)	17.9 (12.9 to 23.7)	13 (11 to 15)	18 (15 to 22)	42.4 (34.6 to 50.8)		15.6 (10.5 to 21.5)
Tracheal, bronchus, and lung cancer	0 (0 to 1)	1 (1 to 1)	47.7 (40.1 to 56.0)	15.0 (10.8 to 19.8)	13 (11 to 15)	18 (15 to 22)	42.4 (34.6 to 50.8)		14.3 (10.1 to 19.2)
Exposure to chromium									
Neoplasms	1 (1 to 1)	2 (1 to 2)	49.3 (42.0 to 56.6)	19.3 (14.2 to 24.4)	27 (24 to 30)	38 (34 to 43)	44.1 (36.8 to 51.2)		17.1 (11.9 to 22.4)
Tracheal, bronchus, and lung cancer	1 (1 to 1)	2 (1 to 2)	49.3 (42.0 to 56.6)	16.3 (12.2 to 20.6)	27 (24 to 30)	38 (34 to 43)	44.1 (36.8 to 51.2)		15.7 (11.6 to 20.0)
Exposure to diesel engine exhaust									
Neoplasms	12 (11 to 13)	18 (16 to 20)	48.9 (41.6 to 55.4)	18.8 (14.1 to 22.9)	344 (304 to 386)	494 (434 to 559)	43.9 (36.8 to 50.5)		16.8 (12.0 to 21.3)
Tracheal, bronchus, and lung cancer	12 (11 to 13)	18 (16 to 20)	48.9 (41.6 to 55.4)	15.8 (12.4 to 18.9)	344 (304 to 386)	494 (434 to 559)	43.9 (36.8 to 50.5)		15.4 (12.0 to 18.7)
Exposure to formaldehyde									
Neoplasms	1 (1 to 1)	1 (1 to 1)	20.6 (13.4 to 28.5)	6.0 (0.9 to 11.7)	40 (33 to 48)	46 (38 to 55)	16.1 (9.4 to 23.6)		5.1 (0.2 to 10.8)
Leukemia	1 (0 to 1)	1 (1 to 1)	18.3 (12.9 to 24.5)	11.5 (7.3 to 16.2)	25 (20 to 30)	28 (23 to 34)	14.5 (9.0 to 20.8)		12.7 (7.2 to 20.5)
Nasopharynx cancer	0 (0 to 1)	0 (0 to 1)	23.9 (10.4 to 39.0)	5.3 (−3.1 to 13.9)	15 (10 to 21)	18 (12 to 25)	18.8 (5.4 to 34.1)		5.1 (−3.5 to 14.3)
Exposure to nickel									
Neoplasms	7 (1 to 18)	9 (1 to 23)	33.9 (25.4 to 55.2)	6.8 (0.1 to 23.8)	184 (26 to 493)	238 (36 to 607)	29.5 (21.2 to 49.1)		4.9 (−2.0 to 21.4)
Tracheal, bronchus, and lung cancer	7 (1 to 18)	9 (1 to 23)	33.9 (25.4 to 55.2)	4.1 (−2.3 to 20.0)	184 (26 to 493)	238 (36 to 607)	29.5 (21.2 to 49.1)		3.7 (−2.8 to 19.0)
Exposure to PAHs									
Neoplasms	3 (3 to 4)	5 (4 to 6)	48.9 (41.4 to 56.4)	19.0 (14.0 to 24.1)	94 (80 to 107)	134 (114 to 156)	43.7 (36.4 to 51.2)		16.8 (11.6 to 22.0)
Tracheal, bronchus, and lung cancer	3 (3 to 4)	5 (4 to 6)	48.9 (41.4 to 56.4)	16.0 (11.7 to 20.2)	94 (80 to 107)	134 (114 to 156)	43.7 (36.4 to 51.2)		15.4 (11.1 to 19.6)
Exposure to silica									
Neoplasms	37 (17 to 58)	49 (22 to 77)	31.2 (24.3 to 41.4)	4.6 (−0.5 to 12.6)	1045 (470 to 1632)	1328 (595 to 2079)	27.1 (20.2 to 36.5)		0.7 (−2.3 to 10.7)
Tracheal, bronchus, and lung cancer	37 (17 to 58)	49 (22 to 77)	31.2 (24.3 to 41.4)	2.0 (−2.7 to 9.4)	1045 (470 to 1632)	1328 (595 to 2079)	27.1 (20.2 to 36.5)		1.7 (−2.9 to 9.0)
Exposure to sulfuric acid									
Neoplasms	3 (1 to 6)	4 (2 to 7)	25.5 (19.2 to 32.4)	0.7 (−4.4 to 6.1)	101 (43 to 182)	123 (53 to 224)	22.5 (16.1 to 29.5)		0.2 (−5.0 to 5.8)
Larynx cancer	3 (1 to 6)	4 (2 to 7)	25.5 (19.2 to 32.4)	4.2 (−0.2 to 8.9)	101 (43 to 182)	123 (53 to 224)	22.5 (16.1 to 29.5)		4.2 (−0.3 to 9.0)
Exposure to trichloroethylene									
Neoplasms	0 (0 to 0)	0 (0 to 0)	51.9 (46.4 to 59.7)	22.0 (17.9 to 27.5)	1 (0 to 2)	2 (0 to 3)	48.8 (43.2 to 56.4)		22.0 (17.7 to 27.7)
Kidney cancer	0 (0 to 0)	0 (0 to 0)	51.9 (46.4 to 59.7)	18.1 (15.7 to 21.0)	1 (0 to 2)	2 (0 to 3)	48.8 (43.2 to 56.4)		19.5 (16.2 to 22.6)

Abbreviations: DALYs, disability-adjusted life-years; PAHs, polycyclic aromatic hydrocarbons; UI, uncertainty interval.

**Figure 1.**
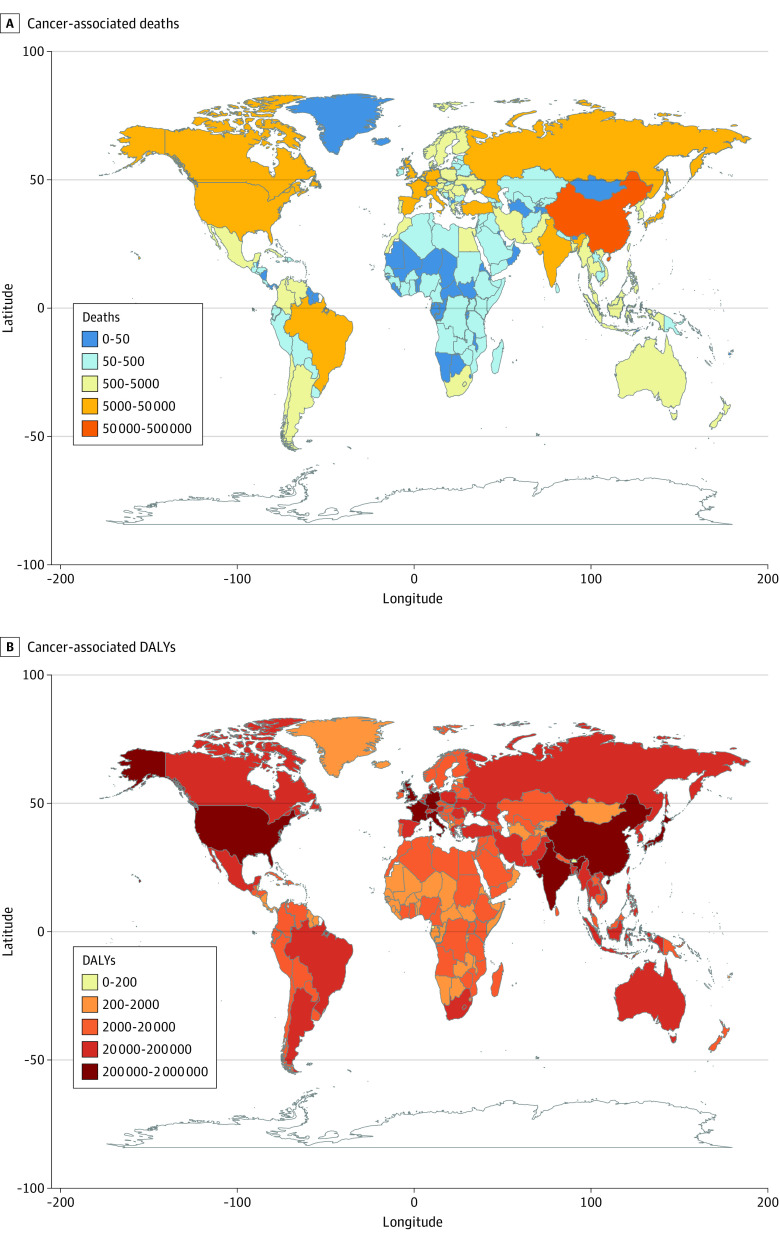
Number of Occupational Carcinogen–Attributable Cancer Deaths and Disability-Adjusted Life-Years (DALYs) for All Ages and Both Sexes in 195 Countries and Territories, 2017 Cancer-associated deaths (A) and DALYs (B).

In 2017, the high SDI region had 175 704 (95% UI, 139 500-210 284) OC-attributable cancer deaths and 2.93 million (95% UI, 2.33 million-3.53 million) cancer DALYs, which was nearly half of the global OC-attributable cancer burden. However, a downward change pattern in age-standardized death and DALY rates was observed between 1990 and 2017. As shown in Figure 1 and eFigure 1 in the Supplement, China (61 644 cancer deaths), the US (42 848), and Japan (20 748) were the top 3 countries with the largest number of OC-attributable cancer deaths in 2017. For DALYs, China (1.47 million), the US (0.71 million), and India (0.37 million) were the 3 countries with the greatest cancer burdens worldwide.

### Cancer Burden Attributable to OCs

Globally, 319 000 (95% UI, 256 000-382 000) cancer deaths were attributable to combined OCs in 2017, which was a 23.7% (95% UI, 20.5%-33.7%) increase from 2007. However, the age-standardized attributable death rate decreased during this period (−2.7%; 95% UI, −5.7% to 0.1%). Disability-adjusted life-years attributable to cancer increased by 21.0% (95% UI, 16.4%-25.0%), and the age-standardized DALY rate decreased (−2.8%; 95% UI, −6.0% to 0.0%). Nine of the 13 OCs were associated with deaths and DALYs of tracheal, bronchial, and lung cancer, and some OCs were associated with other types of cancer. Specifically, exposure to asbestos was attributed to the burden of larynx cancer, mesothelioma, and ovarian cancer. In addition, exposure to benzene was associated with leukemia and nasopharynx cancer, exposure to formaldehyde was associated with laryngeal cancer, and exposure to sulfuric acid was associated with kidney cancer.

### Risk According to Sex and Age

**Figure 2.**
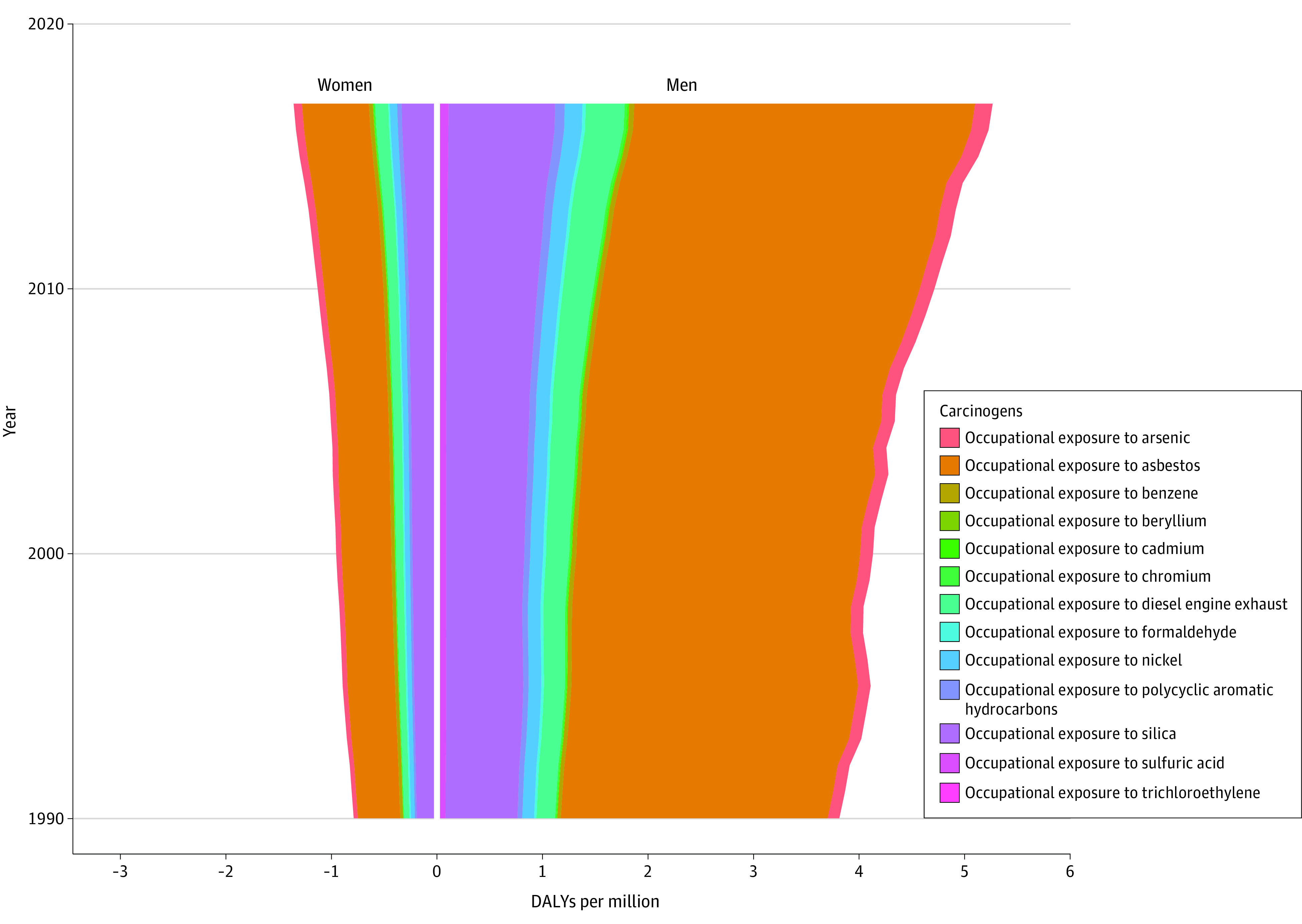
Comparison of Changes in Number of Attributable Cancer Disability-Adjusted Life-Years (DALYs) From 1990 to 2017 for Each of the 13 Occupational Carcinogens Between Men and Women

The relative ranking and attributable burden of OCs varied according to sex and age (Figure 2; eFigure 2 in the Supplement). As the leading OC for both sexes, exposure to asbestos accounted for 3.23 million (95% UI, 2.34 million-4.19 million) cancer DALYs in men and 0.63 million (95% UI, 0.51 million-0.77 million) DALYs in women. The leading 3 OCs (asbestos, silica, and diesel engine exhaust) and the bottom 4 OCs (chromium, cadmium, beryllium, and trichloroethylene) were the same for cancer DALYs in both sexes, and the remaining 6 OC rankings were slightly different. Among the remaining OCs, the most obvious difference was that exposure to sulfuric acid accounted for more DALYs in men (ranked sixth) 7 than in women (ranked ninth) in 2017. Most OC-attributable DALYs in both sexes increased from 1990 to 2017, with the exception of formaldehyde exposure in women, which, after an 8-year increase, began to decrease from 1998 and then switched to an upward change pattern again after 2006.

Globally, the number of OC-attributable cancer deaths and DALYs was the highest in populations older than 70 years, with an upward change pattern from 1990 to 2017, and the lowest among those aged 15 to 49 years (eFigure 2 and eFigure 3 in the Supplement). Occupational carcinogen–associated cancer deaths in the high SDI region was increased more than the deaths in other SDI regions, and the number of attributable deaths and DALYs decreased from 1990 to 2017 in people aged 50 to 69 years. In the other 4 SDI regions, the ranking of age groups according to the highest number of deaths was as follows: ages 50 to 69 years, older than 70 years, and ages 15 to 49 years.

### Changes in Regionally Leading OCs 

The number of total OC-attributable cancer DALYs increased between 1990 and 2017, but the age-standardized DALY rate decreased (eFigure 4 in the Supplement). In 1990, Western Europe (DALYS: 1.52 million; 95% UI, 1.17 million-1.84 million), East Asia (0.62 million; 95% UI, 0.46 million-0.81 million), and Eastern Europe (0.25 million; 95% UI, 0.18-0.34 million) were the 3 regions with the highest burden of OC-attributable cancer DALYs. The leading 3 regions with the highest OC cancer burden in 2017 were East Asia (DALYs: 1.54 million; 95% UI, 1.13 million-2.00 million), Western Europe (1.50 million; 95% UI, 1.21 million-1.79 million), and South Asia (0.50 million; 95% UI, 0.38 million-0.62 million). As for the leading OC, exposure to asbestos ranked first in most of the 21 GBD regions. In 1990, silica was the leading OC in East Asia (39.1% [95% UI, 24.1%-46,5%] of the total risk-attributable DALYs) and Central Asia (35.4% [95% UI, 21.2%-42.8%]). However, in 2017, silica was the leading OC only in East Asia, and asbestos was the leading OC in the other 20 GBD regions. Regarding cancer deaths (eFigure 5 in the Supplement), asbestos exposure was the leading carcinogen worldwide from 1990 to 2017, followed by silica.

### Risk Development and Transformation

The evolution of summary exposure values by region for the 13 OCs in terms of cancer DALYs with SDI changes and the expected summary exposure value level based on SDI alone are shown in eFigure 6 in the Supplement. For most carcinogens, the summary exposure values increased, with SDI values ranging from 0.2 to 0.6, and then decreased, with SDI values from 0.6 to 0.8, and continued to decrease as SDI values increased. General positive associations between summary exposure values and SDI values were observed after exposure to asbestos and arsenic. For exposure to polycyclic aromatic hydrocarbons, summary exposure values increased, with SDI values from 0.2 to 0.8, and then decreased, with SDI values higher than 0.8. These patterns mirrored the complex and profound changes in OC exposure accompanied by changes in social development.

## Discussion

Our study suggests that OC exposure is accountable for global cancer deaths and DALYs and that the relative cancer burden varies greatly across regions depending on age, sex, and social development. For each OC, the respective attributable cancer burden also varied widely, with exposure to asbestos and silica associated with the heaviest cancer burden worldwide. Tracheal, bronchial, and lung cancer were the principal outcomes for most carcinogens.

Overall, there were 13 OCs involved in this research associated with 7 cancer outcomes based on GBD 2017. Consistent with previous studies, lung cancer was the most common cancer type affected by OCs, and asbestos was responsible for the largest number of deaths and DALYs. Dissimilarly, this study was based on GBD 2017 data vs earlier GBD reports in other studies, in which smoking and secondhand smoke were recognized as behavioral risks instead of OCs. Except for that difference, our research appears to be the most comprehensive study to date, and the summary exposure values of these OCs and the variation pattern in different regions and countries were emphasized in our study, which is conducive to exposure control and estimating the probable future cancer burden. Studies have reported that such OC exposures persist in several high-income countries,^13,14,15,16^ particularly exposure to asbestos, silica, and diesel engine exhaust. In addition, OC exposures in medium-low income countries might be even less controlled and more prevalent owing to the lack of automatic monitoring equipment and self-protection at work.^17,18^

Over the past 3 decades, several high-income countries have taken steps to minimize OC exposure to asbestos. The asbestos industry continued to grow throughout the 20th century, first in Western Europe and then in low-income countries during the decade before the 2010s.^5^ However, research in 1960 showed that exposure to asbestos was associated with the development of malignant tumors.^19^ Since then, European countries have spent a long time restricting the production and transportation of asbestos and controlling the demolition of asbestos-containing buildings.^20,21^ Although measures prohibiting the disposal of blue asbestos were implemented in several European countries during this period, the use of asbestos was not fully banned from 1960 to 1993. Furthermore, even with complete cessation of asbestos exposure, asbestos-attributable cancer deaths are expected to continue for another 4 to 5 decades.^2^

The cancer burden attributable to exposure to silica was second to that from asbestos. There was an increase in both the number of cases and age-standardized rates from 2007 to 2017. Silica is widely found in nature, and silica-containing materials are often used, making it among the most prevalent OC exposures.^22^ Workers might be exposed to silica in various production activities, including mining and quarrying, potteries or ceramics, foundries, and various tasks in construction and manufacturing.^23^ Epidemiologic studies on the association between silica exposure and lung cancer are mainly industry based, including mines, quarries, and granite production sites. A quantitative study on the association between silica exposure and lung cancer mortality, which recruited a cohort of 58 677 German uranium miners from 1946 to 2003, confirmed an association between silica exposure and lung cancer, particularly for high exposures (exposure limit >10 mg/m^3^).^24^ A Chinese cohort study^25^ reported that long-term exposure to low levels of silica (exposure limit ≤0.05, ≤0.10, or ≤0.35 mg/m^3^) was associated with an increased risk of total and cause-specific mortality (lung cancer: hazard ratio, 1.08; 95% CI, 1.02-1.14). Therefore, in practice, the need to control the level of silica in the air and use self-protection devices should be emphasized.

Exposure to asbestos and silica mainly increased the burden of tracheal, bronchial, and lung cancer; other risk-outcome pairs were also analyzed. For example, the association between asbestos exposure and malignant mesothelioma, particularly malignant pleural mesothelioma, has been reported.^26,27^ However, owing to the long time that it takes for malignant mesothelioma to develop (30-40 years), we would not see a possible reduction in this health burden at this time and expect a decrease in the number of new cases in the near future, at least in high-income countries.^28^ Attributable OCs for leukemia included benzene and formaldehyde. Occupational benzene exposure could occur in many industries, such as petroleum, chemical production, and manufacturing, and even in shoe-making, painting, printing, and rubber manufacturing.^29,30^ Formaldehyde is an important economic chemical, and more than 2 million workers in the US have access to formaldehyde at work.^31^ Increasing numbers of people are exposed to formaldehyde because it is a component of tobacco smoke and can be released from household products, such as furniture, particleboard, and carpets.

### Limitations

This study has limitations. The main issues relevant to the carcinogen analysis include the exclusion of some carcinogen exposures associated with skin cancer, such as UV exposure from sunlight, and the limited epidemiologic evidence for some cancers. Furthermore, possible unrecognized OCs, mismatching the risk-outcome pairs, exposure to multiple occupational risk factors, and interactions between occupational and other risk factors were not explicitly considered. In addition, analyzing the outcomes of each OC separately may lead to an overestimation of attributable burden, and considering multiple exposures together and treating each exposure as the first to be removed may have led to underestimation.

## Conclusions

In this study, occupational carcinogen exposure appeared to have led to a serious global cancer burden. Among the 13 OCs included in the 2017 GBD study, exposure to asbestos and silica accounted for a large proportion of OC-attributed cancer burden in the past 20 years. Although the average levels of exposure to carcinogens have been reduced, the overall cancer burden attributed to OCs has increased because it mainly reflects past exposure hazards. Our findings identified the cancer burden attributable to OCs based on sex, age, location, and social development. The results presented herein may also provide guidance for the prevention and control programs for OC exposure to carcinogens.

## Notes

Supplement. **eFigure 1.** Attributable Cancer DALYs for Each Occupational Carcinogen for Both Sexes Globally, in Five SDI Quintiles and in 195 Countries and Territories **eFigure 2.** The Proportion of the Three Age Groups for Occupational Carcinogens Attributable Cancer Deaths Globally and in Five SDI Quintiles, 1990-2017 **eFigure 3.** The Proportion of the Three Age Groups for Occupational Carcinogens Attributable Cancer DALYs Globally and in Five SDI Quintiles, 1990-2017 **eFigure 4.** The Comparison of Occupational Carcinogens Attributable Cancer DALYs Between 1990 and 2017, Both Genders, Globally and by GBD Regions **eFigure 5.** The Proportion of Each Occupational Carcinogen Attributable Cancer Deaths Globally and by Regions, Comparison Between 1990 and 2017 **eFigure 6.** Covariation of Summary Exposure Value and Social Demographic Index for 6 of the 13 Occupational Carcinogens in Terms of Attributable Cancer Disability-Adjusted Life-Years (DALYs) in 2017 **eTable 1.** Global Burden of Disease Study (GBD) Risk Factor Hierarchy **eTable 2.** Descriptive Catalogue of the Epidemiological Evidence Used to Assess Whether Each Risk-Outcome Pair Meets the Causal Criteria for Inclusion in the Global Burden of Disease Study 2017 **eTable 3.** Relative Risks Used by Sex for Each Cancer Outcome for All Occupational Carcinogens **eTable 4.** All-Age Deaths, Death PAFs, DALYs, DALY PAFs for Occupational Carcinogens and Cancer Outcome for Both Sexes Combined in 2017 for Global
