# Supplementary material for: Association of 13 Occupational Carcinogens in Patients With Cancer, Individually and Collectively, 1990-2017
Source: JAMA Netw Open. 2021 Feb 18;4(2):e2037530. doi: 10.1001/jamanetworkopen.2020.37530 (PMC7893501; doi:10.1001/jamanetworkopen.2020.37530)
Supplement: Supplement. — eFigure 1. Attributable Cancer DALYs for Each Occupational Carcinogen for Both Sexes Globally, in Five SDI Quintiles and in 195 Countries and Territories eFigure 2. The Proportion of the Three Age Groups for Occupational Carcinogens Attributable Cancer Deaths Globally and in Five SDI Quintiles, 1990-2017 eFigure 3. The Proportion of the Three Age Groups for Occupational Carcinogens Attributable Cancer DALYs Globally and in Five SDI Quintiles, 1990-2017 eFigure 4. The Comparison of Occupational Carcinogens Attributable Cancer DALYs Between 1990 and 2017, Both Genders, Globally and by GBD Regions eFigure 5. The Proportion of Each Occupational Carcinogen Attributable Cancer Deaths Globally and by Regions, Comparison Between 1990 and 2017 eFigure 6. Covariation of Summary Exposure Value and Social Demographic Index for 6 of the 13 Occupational Carcinogens in Terms of Attributable Cancer Disability-Adjusted Life-Years (DALYs) in 2017 eTable 1. Global Burden of Disease Study (GBD) Risk Factor Hierarchy eTable 2. Descriptive Catalogue of the Epidemiological Evidence Used to Assess Whether Each Risk-Outcome Pair Meets the Causal Criteria for Inclusion in the Global Burden of Disease Study 2017 eTable 3. Relative Risks Used by Sex for Each Cancer Outcome for All Occupational Carcinogens eTable 4. All-Age Deaths, Death PAFs, DALYs, DALY PAFs for Occupational Carcinogens and Cancer Outcome for Both Sexes Combined in 2017 for Global [file jamanetwopen-e2037530-s001.pdf]

Supplementary Online Content

Li N, Zhai Z, Zheng Y, et al. Association of 13 occupational carcinogens in patients with cancer, individually and collectively, 1990-2017. *JAMA Netw Open*. 2021;4(2):e2037530. doi:10.1001/jamanetworkopen.2020.37530

eFigure 1. Attributable Cancer DALYs for Each Occupational Carcinogen for Both Sexes Globally, in Five SDI Quintiles and in 195 Countries and Territories

eFigure 2. The Proportion of the Three Age Groups for Occupational Carcinogens Attributable Cancer Deaths Globally and in Five SDI Quintiles, 1990-2017

eFigure 3. The Proportion of the Three Age Groups for Occupational Carcinogens Attributable Cancer DALYs Globally and in Five SDI Quintiles, 1990-2017

eFigure 4. The Comparison of Occupational Carcinogens Attributable Cancer DALYs Between 1990 and 2017, Both Genders, Globally and by GBD Regions

eFigure 5. The Proportion of Each Occupational Carcinogen Attributable Cancer Deaths Globally and by Regions, Comparison Between 1990 and 2017

eFigure 6. Covariation of Summary Exposure Value and Social Demographic Index for 6 of the 13 Occupational Carcinogens in Terms of Attributable Cancer Disability-Adjusted Life-years (DALYs) in 2017

eTable 1. Global Burden of Disease Study (GBD) Risk Factor Hierarchy

eTable 2. Descriptive Catalogue of the Epidemiological Evidence Used to Assess Whether Each Risk-Outcome Pair Meets the Causal Criteria for Inclusion in the Global Burden of Disease Study 2017

eTable 3. Relative Risks Used by Sex for Each Cancer Outcome for All Occupational Carcinogens

eTable 4. All-Age Deaths, Death PAFs, DALYs, DALY PAFs for Occupational Carcinogens and Cancer Outcome for Both Sexes Combined in 2017 for Global

This supplementary material has been provided by the authors to give readers additional information about their work.

© 2021 Li, N, et al. *JAMA Network Open.*

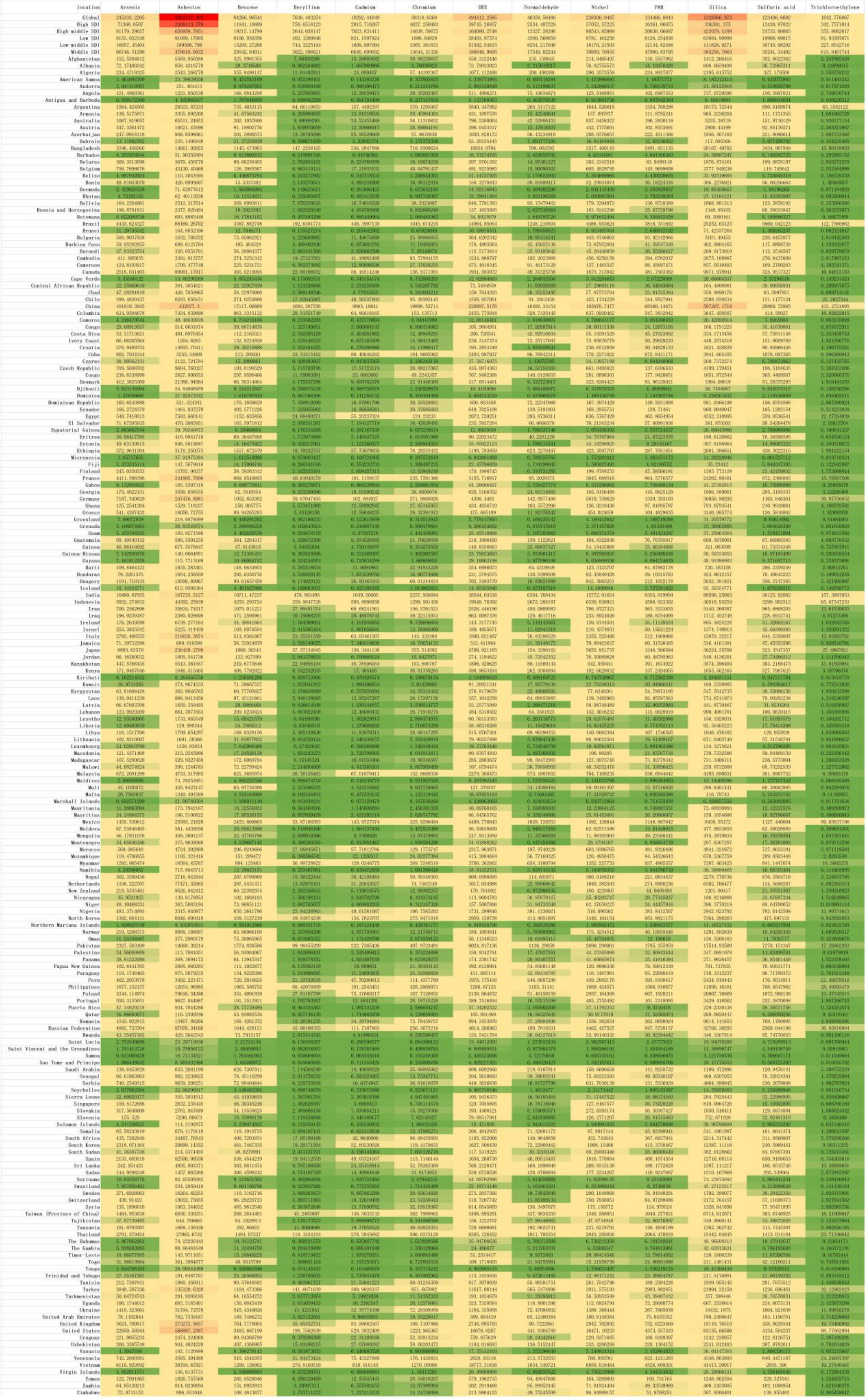

eFigure 1. Attributable cancer DALYs for each occupational carcinogen for both sexes, 2017, globally, in five SDI quintiles and in 195 countries and territories. Abbreviations: DEE, diesel engine exhaust; PAH, polycyclic aromatic hydrocarbon; DALYs, disability-adjusted life years; SDI, social development index.

© 2021 Li, N, et al. *JAMA Network Open*.

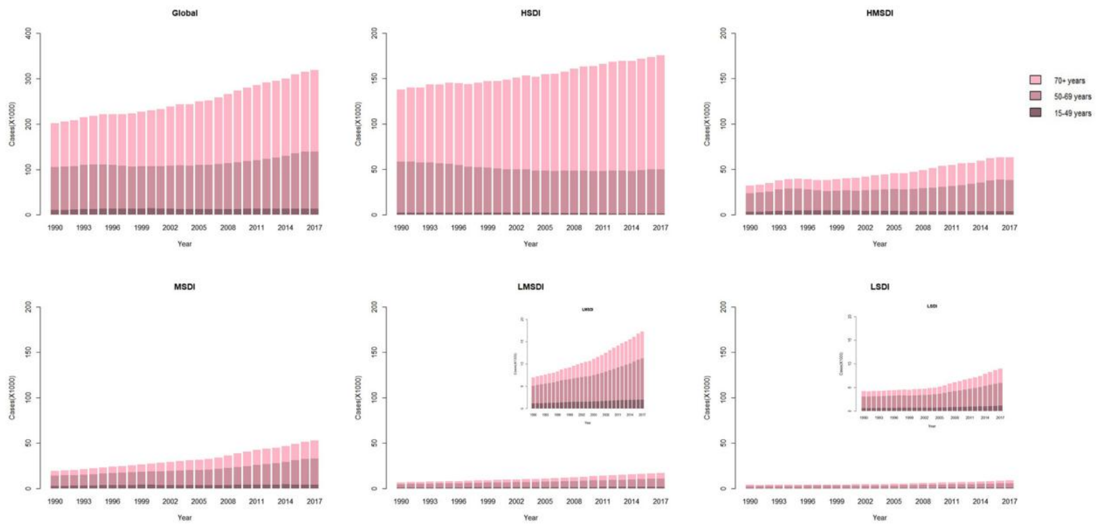

eFigure 2. The proportion of the three age groups for occupational carcinogens attributable cancer deaths globally and in five SDI quintiles, 1990-2017. The populations were divided into three age groups: 15-49 years, 50-69 years, and 70+ years. And the five SDI quintiles included high, high-middle, middle, low-middle, and low SDI regions. Abbreviation: SDI, social development index.

© 2021 Li, N, et al. *JAMA Network Open.*

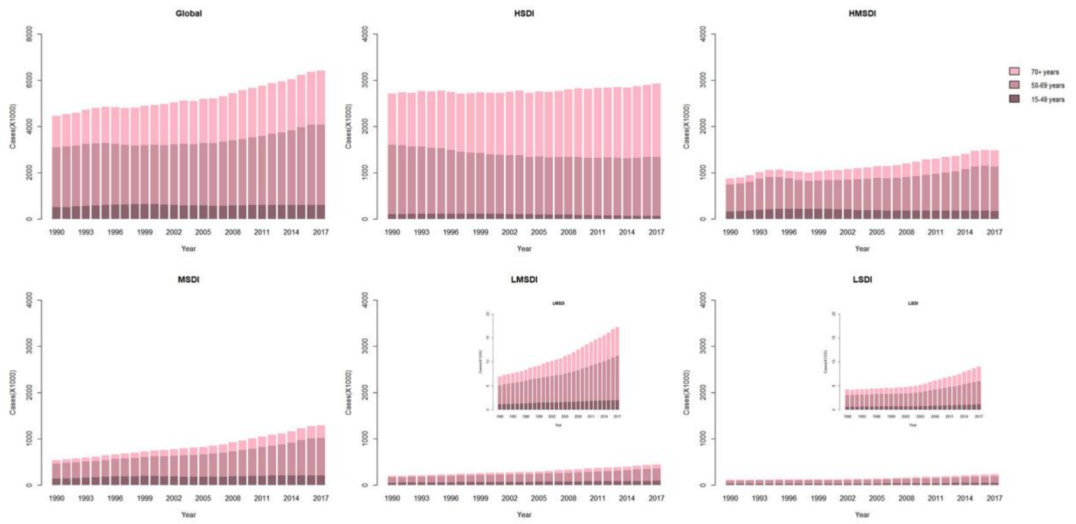

eFigure 3. The proportion of the three age groups for occupational carcinogens attributable cancer DALYs globally and in five SDI quintiles, 1990-2017. The populations were divided into three age groups: 15-49 years, 50-69 years, and 70+ years. And the five SDI quintiles included high, high-middle, middle, low-middle, and low SDI regions. Abbreviations: SDI, social development index; DALYs, disability-adjusted life years.

© 2021 Li, N, et al. *JAMA Network Open.*

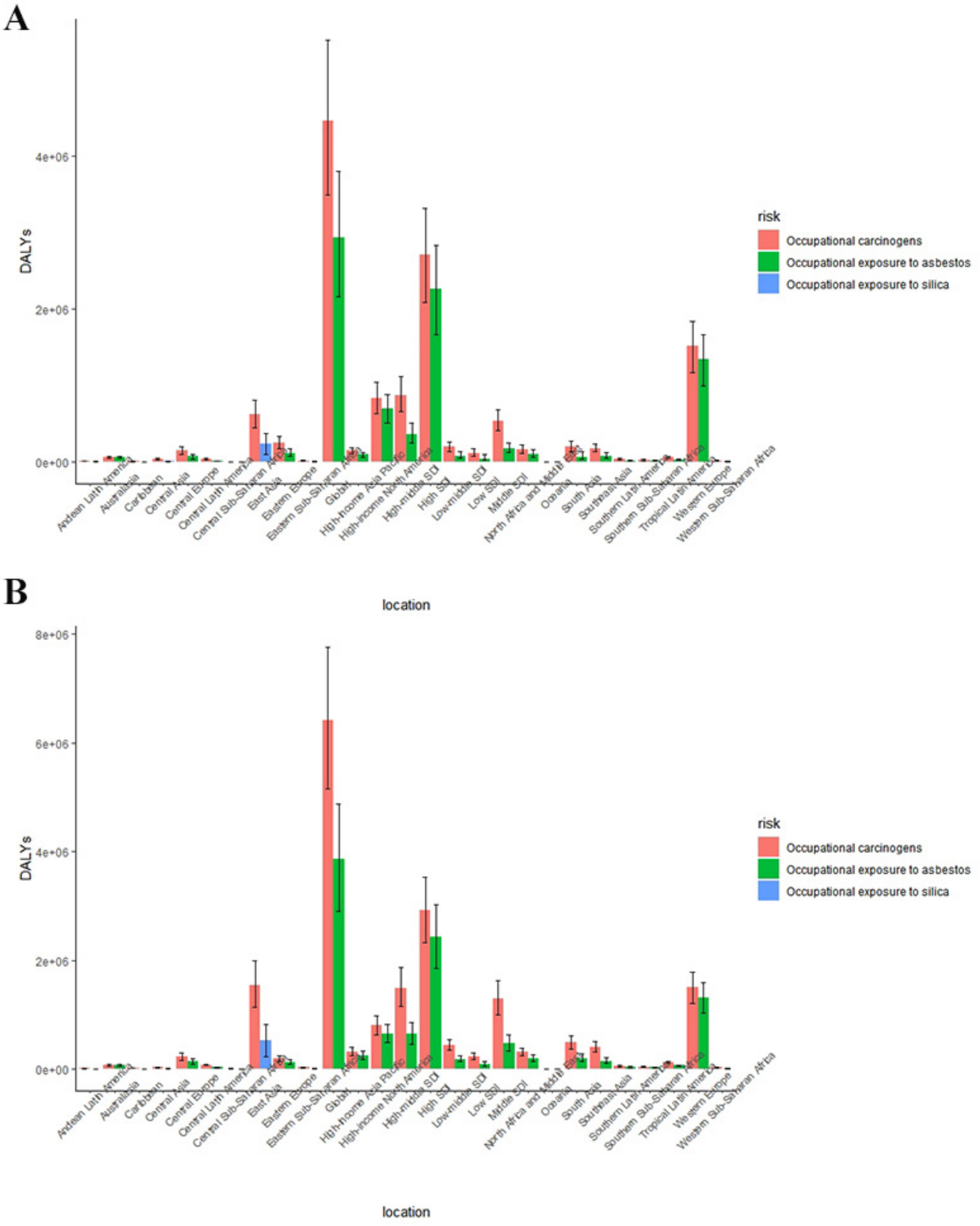

eFigure 4. The comparison of occupational carcinogens attributable cancer DALYs between 1990 and 2017, both genders, globally and by GBD regions. Only the overall cancer DALYs and the most pronounced one attributable to specific occupational risk factors were presented. (A) attributable cancer DALYs in 1990; (B) attributable cancer DALYs in 2017. Abbreviations: GBD, Global Burden of Disease; DALYs, disability adjusted life years.

© 2021 Li, N, et al. *JAMA Network Open.*

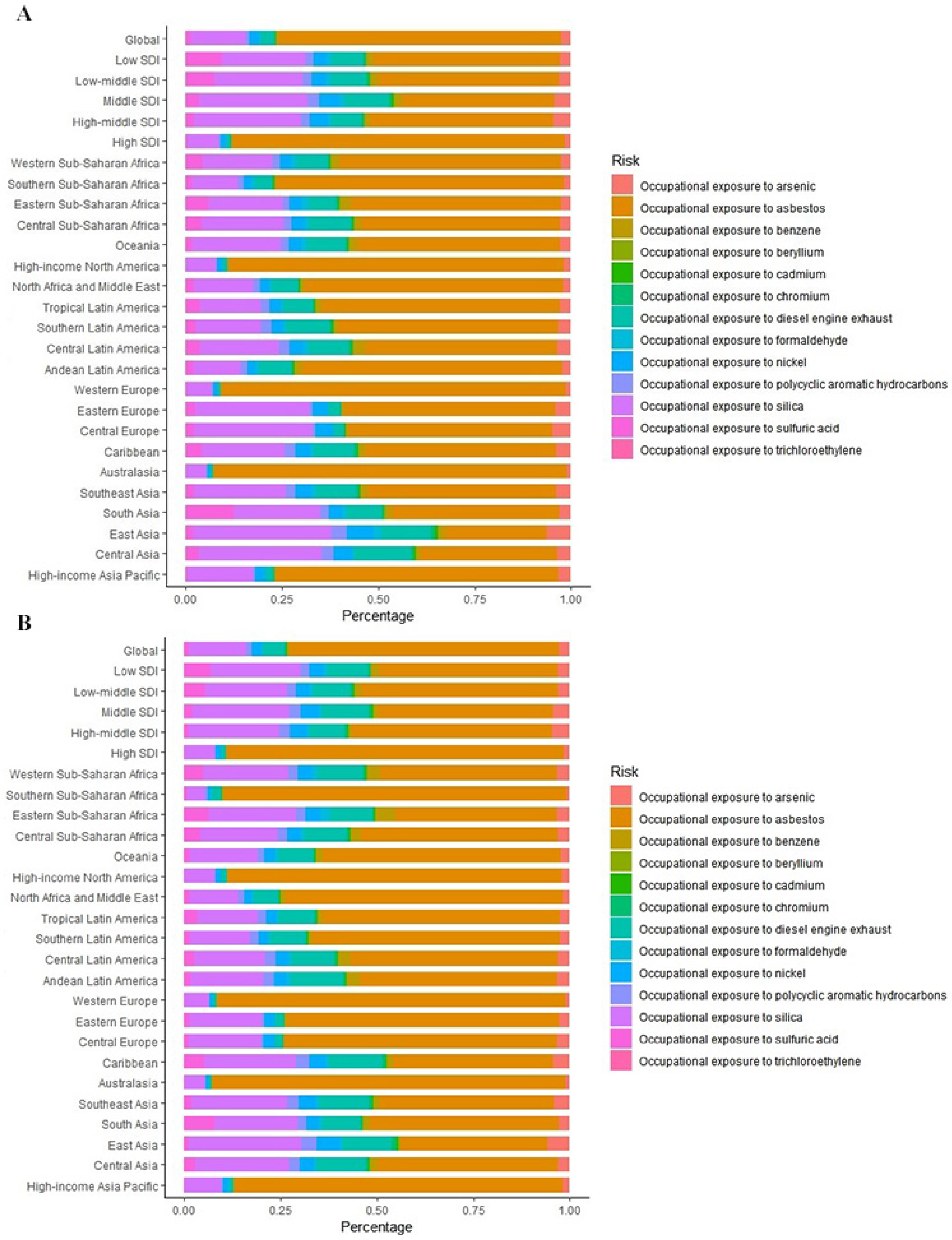

eFigure 5. The proportion of each occupational carcinogen attributable cancer deaths globally and by regions, comparison between 1990 and 2017. (A) proportion of attributable cancer deaths in 1990; (B) proportion of attributable cancer deaths in 2017.

© 2021 Li, N, et al. *JAMA Network Open.*

eFigure 6. Covariation of Summary Exposure Value and Social Demographic Index for 6 of the 13 Occupational Carcinogens in Terms of Attributable Cancer Disability-Adjusted Life-years (DALYs) in 2017

6A

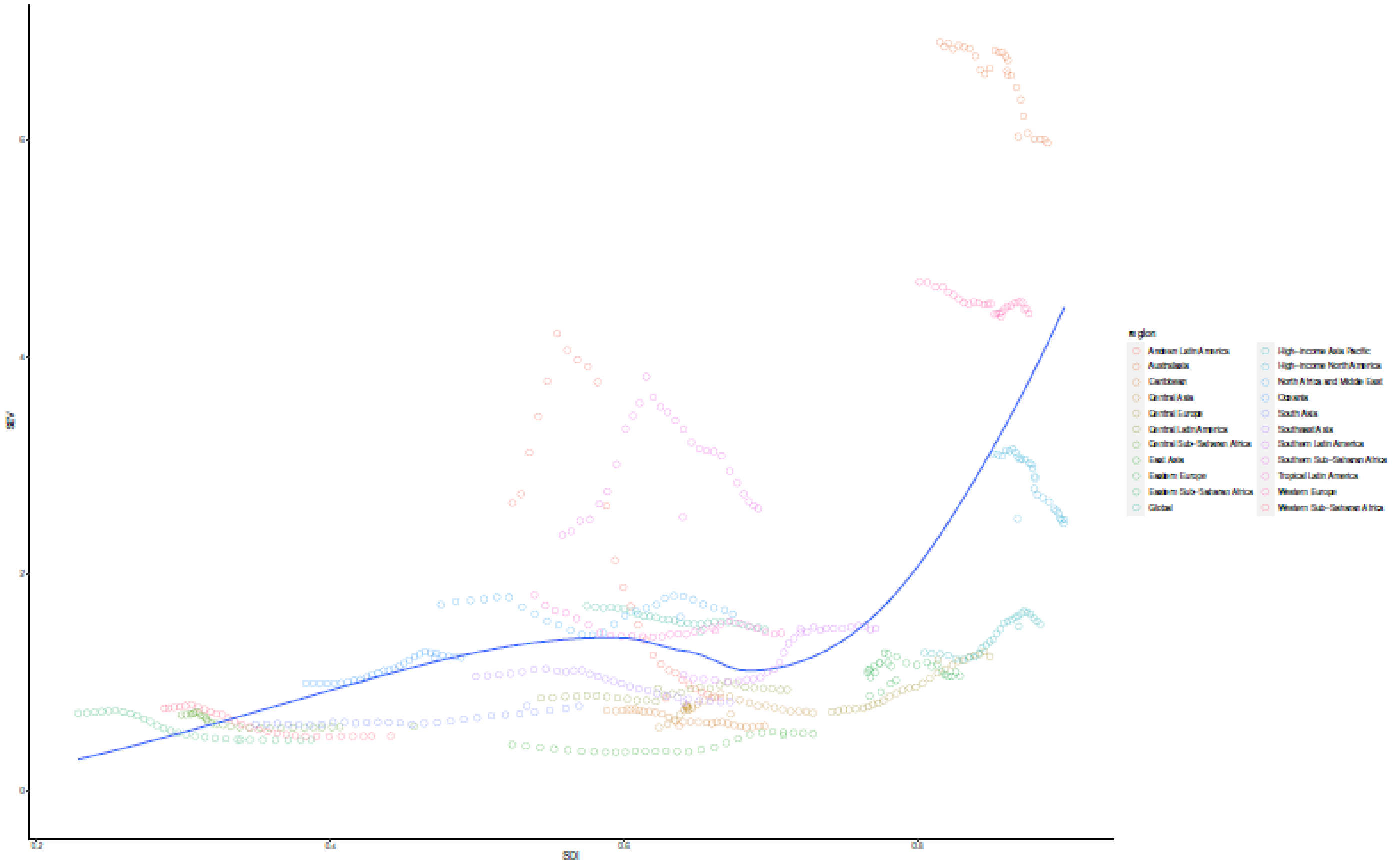

© 2021 Li, N, et al. *JAMA Network Open.*

6B

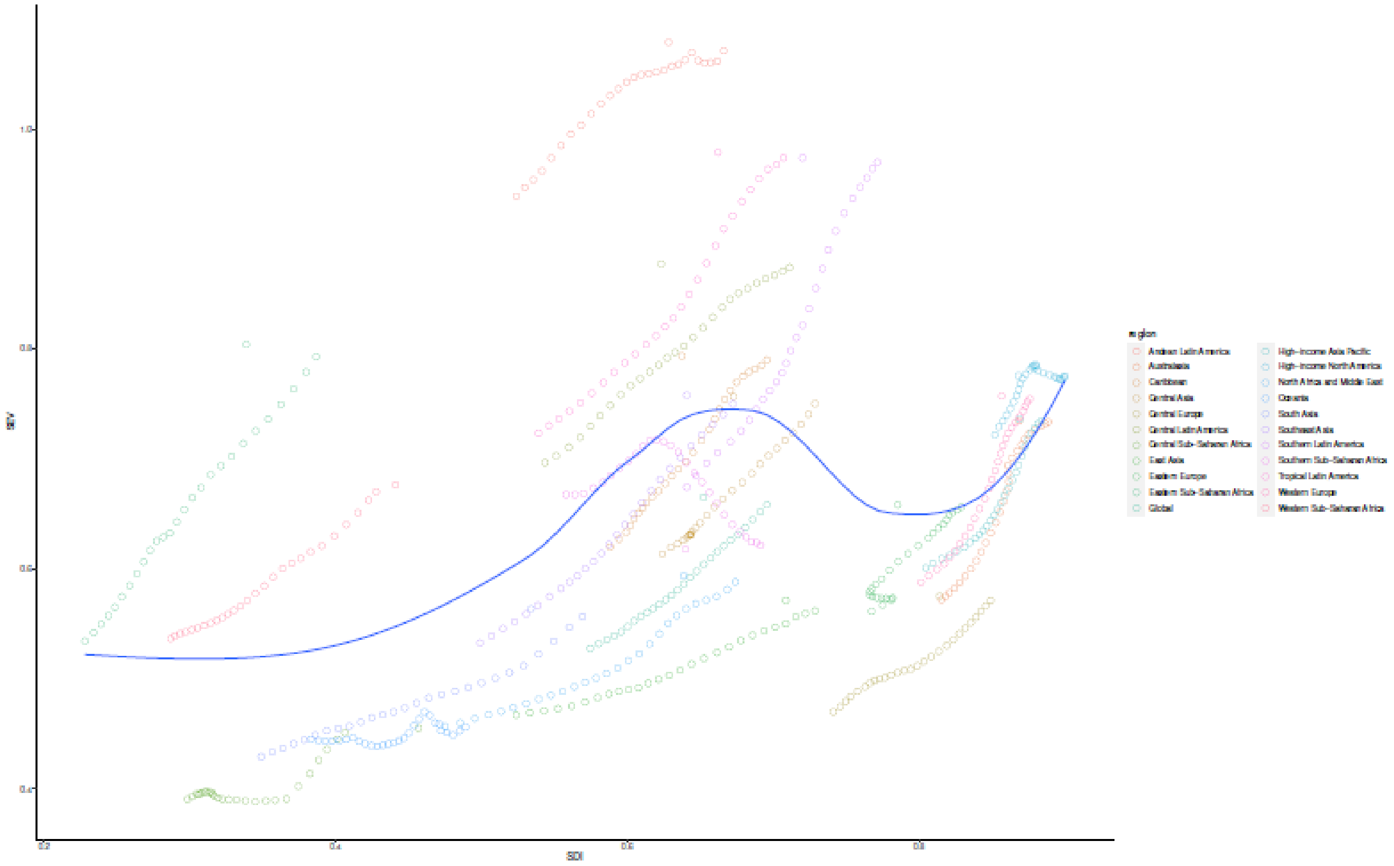

© 2021 Li, N, et al. *JAMA Network Open.*

6C

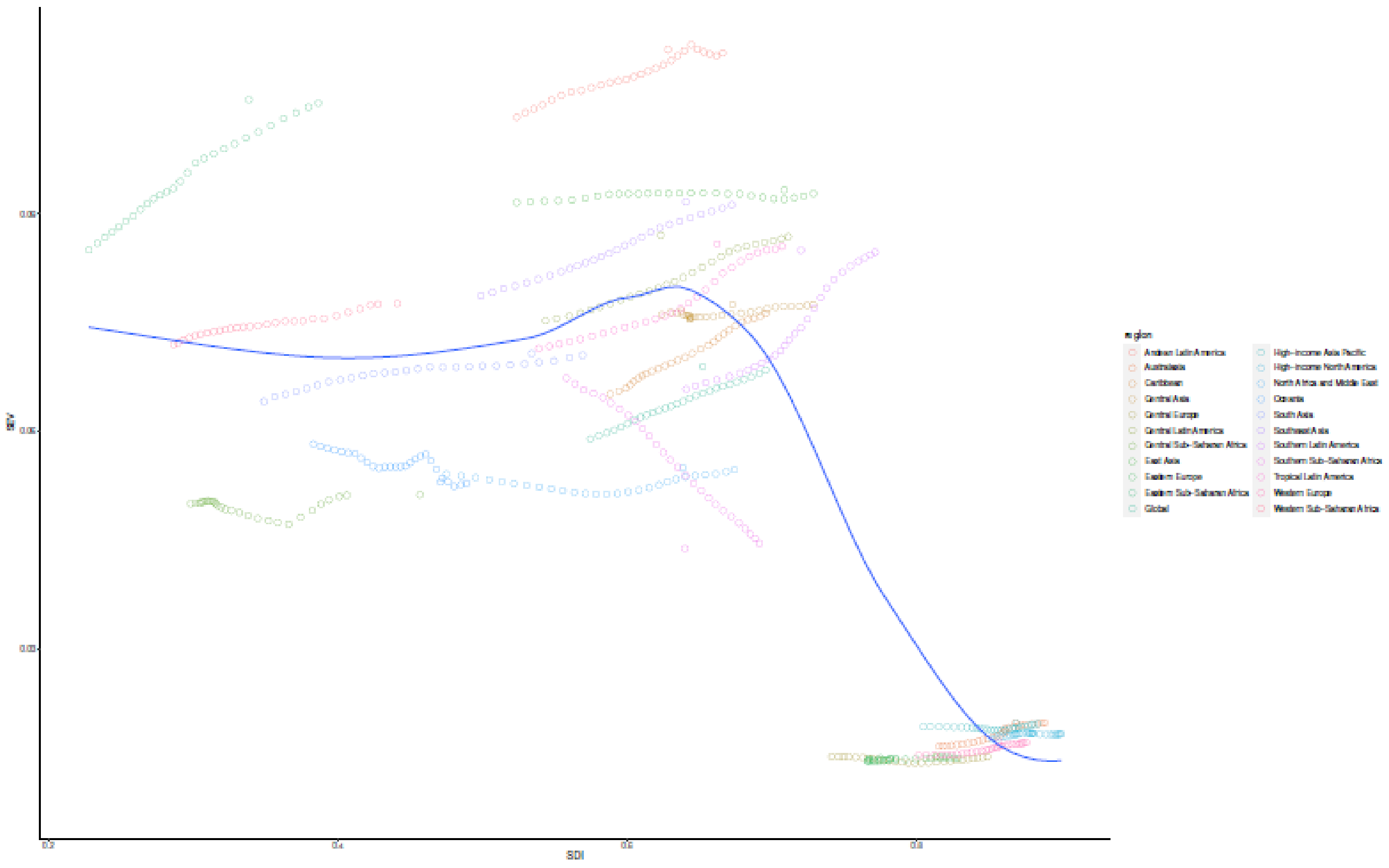

© 2021 Li, N, et al. *JAMA Network Open.*

6D

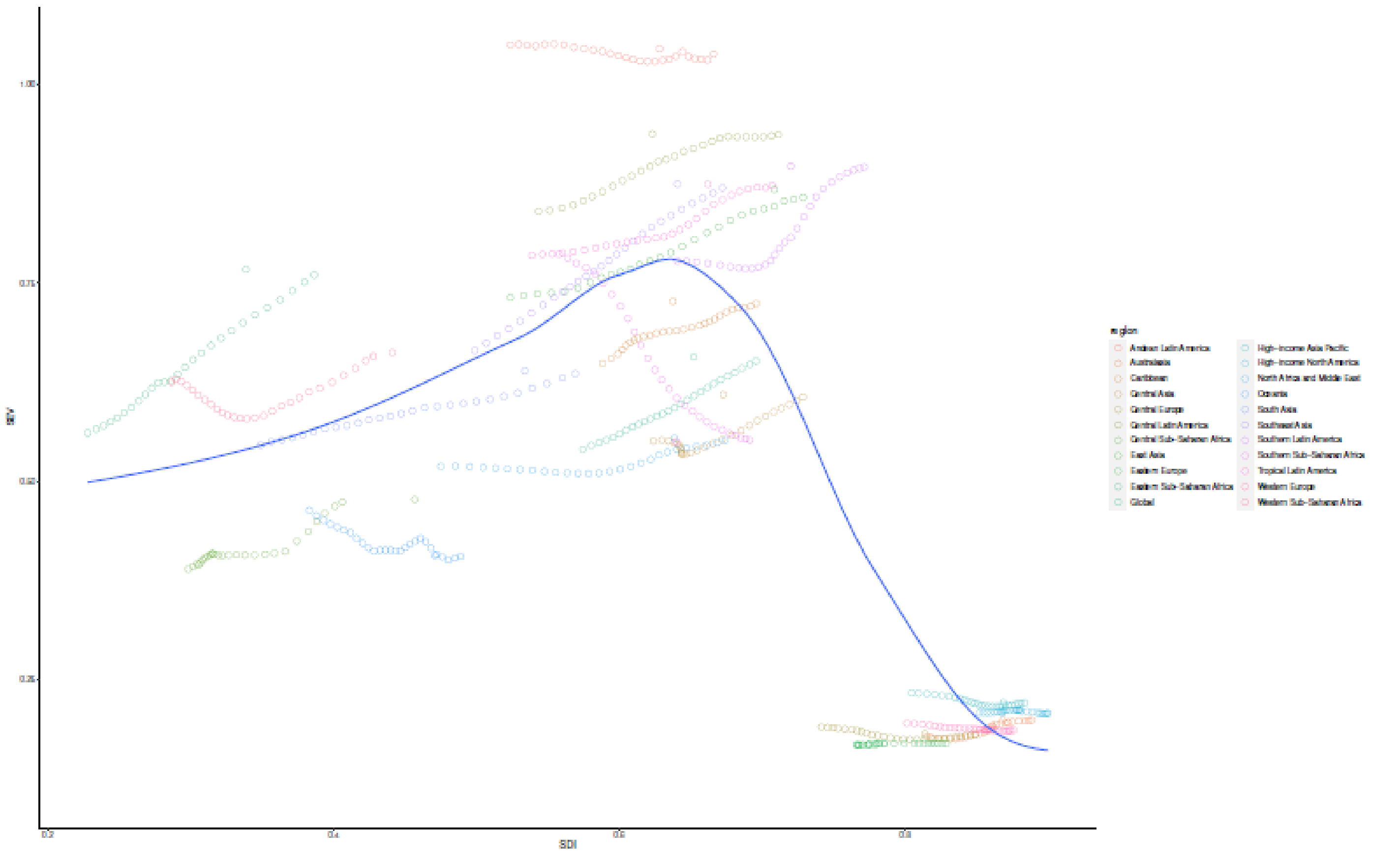

© 2021 Li, N, et al. *JAMA Network Open.*

6E

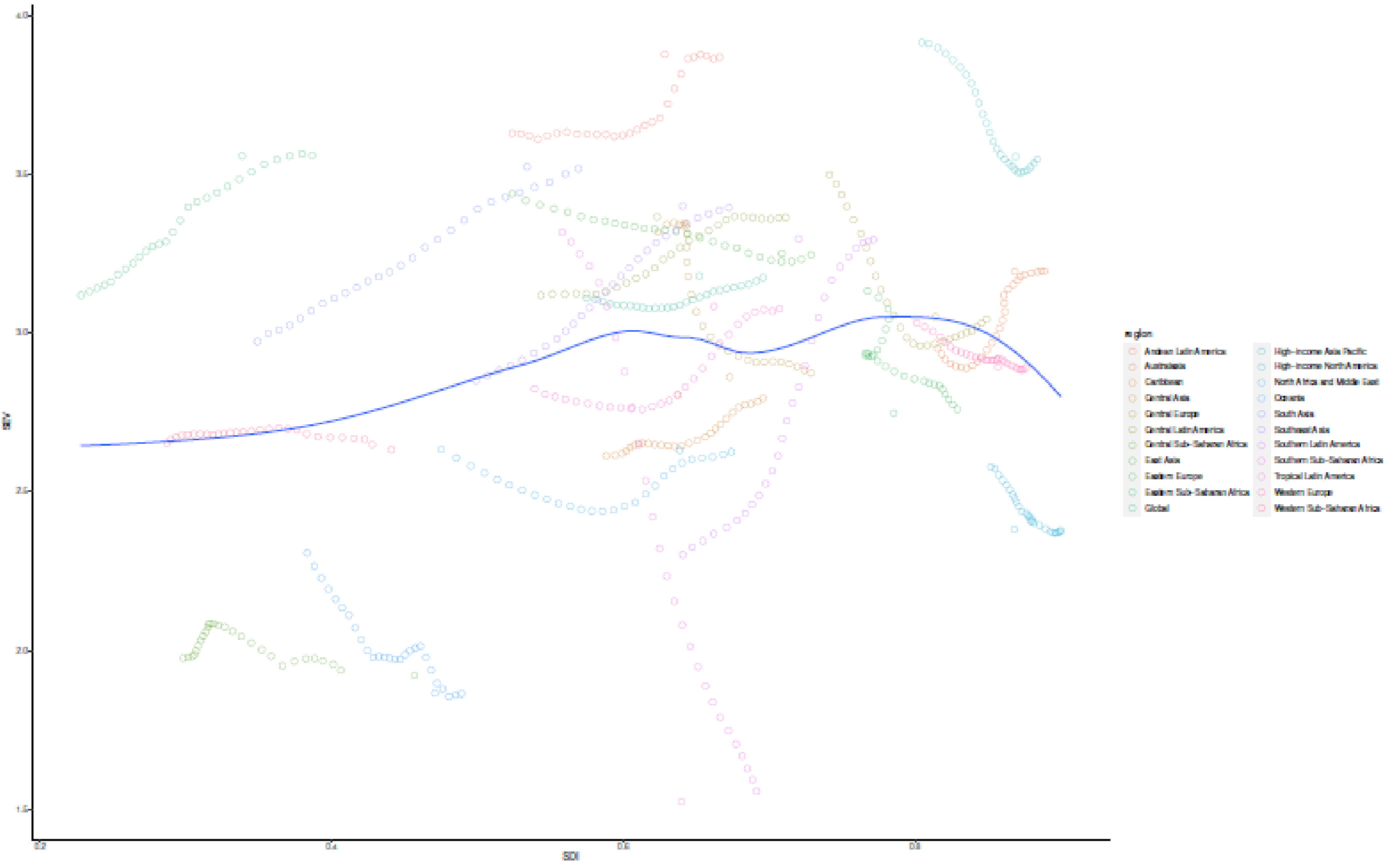

© 2021 Li, N, et al. *JAMA Network Open.*

6F

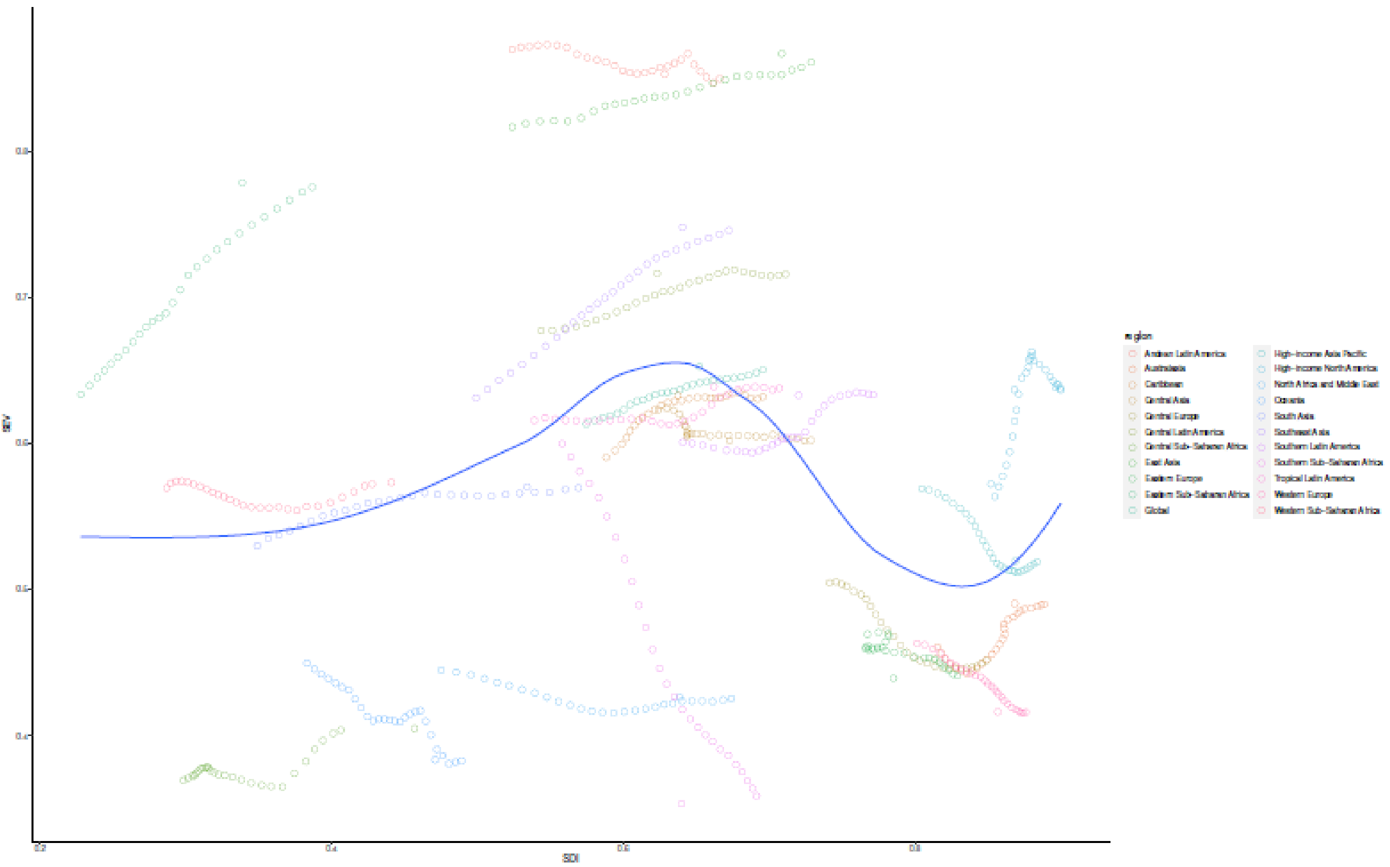

© 2021 Li, N, et al. *JAMA Network Open.*

eTable 1. Global Burden of Disease Study (GBD) risk factor hierarchy.

Level 0	Level 1	Level 2	Level 3	Level 4
**All risk factors**	**Environmental/occupational risk factors**	Unsafe water, sanitation, and handwashing	Unsafe water source	
			Unsafe sanitation	
			No access to handwashing facility	
		Air pollution	Particulate matter pollution	Ambient particulate matter pollution
				Household air pollution from solid fuels
			Ambient ozone pollution	
		Other environmental risks	Residential radon	
			Lead exposure	
		Occupational risks	Occupational carcinogens	Occupational exposure to asbestos
				Occupational exposure to arsenic
				Occupational exposure to benzene
				Occupational exposure to beryllium
				Occupational exposure to cadmium
				Occupational exposure to chromium
				Occupational exposure to diesel engine exhaust
				Occupational exposure to formaldehyde
				Occupational exposure to nickel
				Occupational exposure to polycyclic aromatic hydrocarbons
				Occupational exposure to silica
				Occupational exposure to sulfuric acid
				Occupational exposure to trichloroethylene
			Occupational asthmagens	
			Occupational particulate matter, gases, and fumes	
			Occupational noise	
			Occupational injuries	

© 2021 Li, N, et al. *JAMA Network Open.*

			Occupational ergonomic factors	
	**Behavioral risks**	Child and maternal malnutrition	Suboptimal breast feeding	Non-exclusive breast feeding
				Discontinued breast feeding
			Child growth failure	Child underweight
				Child wasting
				Child stunting
			Low birth weight and short gestation	Short gestation for birth weight
				Low birth weight for gestation
			Iron deficiency<br>Vitamin A deficiency<br>Zinc deficiency	
		Tobacco	smoking<br>Chewing tobacco<br>Secondhand smoke	
		Alcohol use<br>Drug use		
		Dietary risks	Diet low in fruits<br>Diet low in vegetables<br>Diet low in legumes<br>Diet low in whole grains<br>Diet low in nuts and seeds<br>Diet low in milk<br>Diet high in red meat<br>Diet high in processed meat<br>Diet high in sugar-sweetened beverages<br>Diet low in fiber<br>Diet low in calcium	

© 2021 Li, N, et al. *JAMA Network Open.*

			Diet low in seafood omega-3 fatty acids<br>Diet low in polyunsaturated fatty acids<br>Diet high in trans fatty acids<br>Diet high in sodium
		Intimate partner violence	
		Childhood maltreatment	Childhood sexual abuse<br>Bullying victimization
		Unsafe sex<br>Low physical activity	
	**Metabolic risks**	High fasting plasma glucose<br>High LDL cholesterol<br>High systolic blood pressure<br>High body-mass index<br>Low bone mineral density<br>Impaired kidney function	

© 2021 Li, N, et al. *JAMA Network Open.*

eTable 2. Descriptive catalogue of the epidemiological evidence used to assess whether each risk-outcome pair meets the causal criteria for inclusion in the Global Burden of Disease Study 2017.

Occupational carcinogens	Outcome	RCTs (n)	RCTs with significant effect in the opposite direction (%)	RCTs with null findings (%)	Prospective observational studies (n)*	Prospective observational studies with significant association in the opposite direction (%)	Case-control studies assessing the risk-outcome pair relationship (n)**	Case-control studies that show significant association in the opposite direction (%)	Lower limit of RR > 1.5	Dose-response relationship	Biological plausibility †	Analogy ‡
Exposure to arsenic	Tracheal, bronchus, and lung cancer	0	-	-	9	0	-	-	No	-	Yes	No
Exposure to asbestos	Larynx cancer	0	-	-	27	0	-	-	No	-	Yes	Yes
Exposure to asbestos	Tracheal, bronchus, and lung cancer	0	-	-	18	0	-	-	Yes	-	Yes	Yes
Exposure to asbestos	Ovarian cancer	0	-	-	15	0	-	-	No	-	Yes	Yes
Exposure to asbestos	Mesothelioma	0	-	-	5	0	-	-	Yes	-	Yes	Yes
Exposure to benzene	Leukemia	0	-	-	12	0	-	-	Yes	-	Yes	No
Exposure to beryllium	Tracheal, bronchus, and lung cancer	0	-	-	3	0	2	0	No	-	Yes	No
Exposure to cadmium	Tracheal, bronchus, and lung cancer	0	-	-	7	0	-	-	No	-	Yes	No
Exposure to chromium	Tracheal, bronchus, and lung cancer	0	-	-	26	0	-	-	No	-	Yes	No
Exposure to diesel engine exhaust	Tracheal, bronchus, and lung cancer	0	-	-	17	0	-	-	No	-	Yes	No
Exposure to	Nasopharynx	0	-	-	2	0	6	0	No	-	Yes	Yes

© 2021 Li, N, et al. *JAMA Network Open.*

formaldehyde	cancer											
Exposure to formaldehyde	Leukemia	0	-	-	13	0	-	-	No	-	Yes	Yes
Exposure to nickel	Tracheal, bronchus, and lung cancer	0	-	-	6	0	-	-	No	-	Yes	No
Exposure to PAHs	Tracheal, bronchus, and lung cancer	0	-	-	39	0	-	-	No	-	Yes	No
Exposure to silica	Tracheal, bronchus, and lung cancer	0	-	-	17	0	-	-	No	-	Yes	No
Exposure to sulfuric acid	Larynx cancer	0	-	-	14	0	-	-	Yes	-	Yes	No
Exposure to trichloroethylene	Kidney cancer	0	-	-	20	0	-	-	No	-	Yes	No

Abbreviation: PAHs, polycyclic aromatic hydrocarbons

© 2021 Li, N, et al. *JAMA Network Open.*

eTable 3. Relative risks used by sex for each cancer outcome for all occupational carcinogens.

Risk - Outcome pairs	Category / Units	Morbidity / Mortality	Sex	Relative Risks of All ages
**Occupational exposure to asbestos**				
Larynx cancer	High vs. Low exposure	Both	Males	1.38 (1.19 to 1.61)
Larynx cancer	High vs. Low exposure	Both	Females	1.39 (1.19 to 1.60)
Tracheal, bronchus, and lung cancer	High vs. Low exposure	Both	Males	2.28 (1.74 to 2.94)
Tracheal, bronchus, and lung cancer	High vs. Low exposure	Both	Females	1.88 (1.59 to 2.21)
Ovarian cancer	High vs. Low exposure	Both	Females	1.81 (1.39 to 2.31)
**Occupational exposure to arsenic**				
Tracheal, bronchus, and lung cancer	High vs. No exposure	Both	Both	2.06 (1.52 to 2.55)
Tracheal, bronchus, and lung cancer	Low vs. No exposure	Both	Both	1.75 (0.70 to 2.78)
**Occupational exposure to benzene**				
Acute lymphoid leukaemia	High vs. No exposure	Both	Both	2.62 (1.22 to 3.98)
Acute lymphoid leukaemia	Low vs. No exposure	Both	Both	1.63 (1.00 to 2.26)
Chronic lymphoid leukaemia	High vs. No exposure	Both	Both	2.62 (1.22 to 3.98)
Chronic lymphoid leukaemia	Low vs. No exposure	Both	Both	1.63 (1.00 to 2.26)
Acute myeloid leukaemia	High vs. No exposure	Both	Both	2.62 (1.22 to 3.98)
Acute myeloid leukaemia	Low vs. No exposure	Both	Both	1.63 (1.00 to 2.26)
Chronic myeloid leukaemia	High vs. No exposure	Both	Both	2.62 (1.22 to 3.98)
Chronic myeloid leukaemia	Low vs. No exposure	Both	Both	1.63 (1.00 to 2.26)
Other leukaemia	High vs. No exposure	Both	Both	2.62 (1.22 to 3.98)
Other leukaemia	Low vs. No exposure	Both	Both	1.63 (1.00 to 2.26)
**Occupational exposure to beryllium**				

© 2021 Li, N, et al. *JAMA Network Open.*

Tracheal, bronchus, and lung cancer	High vs. Low or No exposure	Both	Males	1.17 (1.09 to 1.30)
Tracheal, bronchus, and lung cancer	High vs. Low or No exposure	Both	Females	1.17 (1.08 to 1.27)
**Occupational exposure to cadmium**				
Tracheal, bronchus, and lung cancer	High vs. Low or No exposure	Both	Males	1.19 (1.10 to 1.29)
Tracheal, bronchus, and lung cancer	High vs. Low or No exposure	Both	Females	1.19 (1.09 to 1.30)
**Occupational exposure to chromium**				
Tracheal, bronchus, and lung cancer	High vs. Low or No exposure	Both	Males	1.18 (1.11 to 1.25)
Tracheal, bronchus, and lung cancer	High vs. Low or No exposure	Both	Females	1.18 (1.12 to 1.25)
**Occupational exposure to diesel engine exhaust**				
Tracheal, bronchus, and lung cancer	High vs. Low or No exposure	Both	Males	1.47 (1.30 to 1.66)
Tracheal, bronchus, and lung cancer	High vs. Low or No exposure	Both	Females	1.47 (1.29 to 1.68)
**Occupational exposure to formaldehyde**				
Nasopharynx cancer	High vs. Low or No exposure	Both	Males	2.22 (1.03 to 4.23)
Nasopharynx cancer	High vs. Low or No exposure	Both	Females	2.20 (1.04 to 4.06)
Acute lymphoid leukaemia	High vs. Low or No exposure	Both	Males	1.48 (1.19 to 1.82)
Acute lymphoid leukaemia	High vs. Low or No exposure	Both	Females	1.49 (1.20 to 1.85)
Chronic lymphoid leukaemia	High vs. Low or No exposure	Both	Males	1.48 (1.19 to 1.82)
Chronic lymphoid leukaemia	High vs. Low or No exposure	Both	Females	1.49 (1.20 to 1.85)

© 2021 Li, N, et al. *JAMA Network Open.*

Acute myeloid leukaemia	High vs. Low or No exposure	Both	Males	1.48 (1.19 to 1.82)
Acute myeloid leukaemia	High vs. Low or No exposure	Both	Females	1.49 (1.20 to 1.85)
Chronic myeloid leukaemia	High vs. Low or No exposure	Both	Males	1.48 (1.19 to 1.82)
Chronic myeloid leukaemia	High vs. Low or No exposure	Both	Females	1.49 (1.20 to 1.85)
Other leukaemia	High vs. Low or No exposure	Both	Males	1.48 (1.19 to 1.82)
Other leukaemia	High vs. Low or No exposure	Both	Females	1.49 (1.20 to 1.85)
**Occupational exposure to nickel**				
Tracheal, bronchus, and lung cancer	High vs. No exposure	Both	Both	2.15 (1.30 to 3.30)
Tracheal, bronchus, and lung cancer	Low vs. No exposure	Both	Both	1.54 (0.61 to 3.36)
**Occupational exposure to polycyclic aromatic hydrocarbons**				
Tracheal, bronchus, and lung cancer	High vs. Low or No exposure	Both	Males	1.31 (1.17 to 1.47)
Tracheal, bronchus, and lung cancer	High vs. Low or No exposure	Both	Females	1.31 (1.15 to 1.49)
**Occupational exposure to silica**				
Tracheal, bronchus, and lung cancer	High vs. No exposure	Both	Both	1.70 (1.16 to 2.26)
Tracheal, bronchus, and lung cancer	Low vs. No exposure	Both	Both	1.54 (1.06 to 1.99)
**Occupational exposure to sulfuric acid**				
Larynx cancer	High vs. No exposure	Both	Both	4.57 (2.12 to 8.33)
Larynx cancer	Low vs. No exposure	Both	Both	2.02 (0.94 to 3.78)
**Occupational exposure to trichloroethylene**				
Kidney cancer	High vs. Low or No exposure	Both	Both	1.25 (1.05 to 1.46)

© 2021 Li, N, et al. *JAMA Network Open.*

eTable 4. All-age deaths, death PAFs, DALYs, DALY PAFs for occupational carcinogens and cancer outcome for both sexes combined in 2017 for Global.

	2017 Deaths (in thousands)	2017 Deaths PAF	2017 DALYs (in thousands)	2017 DALYs PAF
**Occupational carcinogens: All cancer outcomes**	334 (271 to 397)	0.6% (0.5% to 0.7%)	6,750 (5,490 to 8,120)	0.3% (0.2% to 0.3%)
**Occupational exposure to asbestos: All cancer outcomes**	232 (177 to 289)	0.4% (0.3% to 0.5%)	3,930 (2,980 to 4,950)	0.2% (0.1% to 0.2%)
Larynx cancer	4 (2 to 6)	3.1% (1.8% to 4.7%)	73 (41 to 112)	2.2% (1.3% to 3.4%)
Tracheal, bronchus, and lung cancer	191 (137 to 247)	10.2% (7.3% to 13.1%)	3,120 (2,180 to 4,110)	7.6% (5.3% to 10.0%)
Ovarian cancer	6 (3 to 9)	3.6% (1.8% to 5.5%)	100 (49 to 156)	2.1% (1.0% to 3.3%)
Mesothelioma	27 (26 to 28)	91.8% (90.0% to 93.4%)	569 (542 to 598)	84.8% (81.9% to 87.5%)
**Occupational exposure to arsenic: All cancer outcomes**	9 (2 to 16)	0.5% (0.1% to 0.9%)	245 (64 to 436)	0.0% (0.0% to 0.0%)
Tracheal, bronchus, and lung cancer	9 (2 to 16)	0.5% (0.1% to 0.9%)	245 (64 to 436)	0.0% (0.0% to 0.0%)
**Occupational exposure to benzene: All cancer outcomes**	1 (0 to 3)	0.0% (0.0% to 0.0%)	84 (25 to 137)	0.0% (0.0% to 0.0%)
Occupational exposure to benzene: Leukemia	1 (0 to 3)	0.5% (0.2% to 0.9%)	84 (25 to 137)	0.7% (0.2% to 1.1%)
Acute lymphoid leukemia	0 (0 to 0)	0.7% (0.2% to 1.1%)	17 (5 to 29)	0.7% (0.2% to 1.1%)
Chronic lymphoid leukemia	0 (0 to 0)	0.3% (0.1% to 0.4%)	3 (1 to 5)	0.5% (0.2% to 0.8%)

© 2021 Li, N, et al. *JAMA Network Open.*

Acute myeloid leukemia	0<br>(0 to 1)	0.6%<br>(0.2% to 0.9%)	25<br>(8 to 41)	0.8%<br>(0.3% to 1.3%)
Chronic myeloid leukemia	0<br>(0 to 0)	0.6%<br>(0.2% to 1.0%)	6<br>(2 to 11)	1.0%<br>(0.3% to 1.7%)
Other leukemia	0<br>(0 to 1)	0.5%<br>(0.2% to 0.8%)	31<br>(9 to 52)	0.7%<br>(0.2% to 1.1%)
**Occupational exposure to beryllium: All cancer outcomes**	0<br>(0 to 0)	0.0%<br>(0.0% to 0.0%)	7<br>(6 to 9)	0.0%<br>(0.0% to 0.0%)
Tracheal, bronchus, and lung cancer	0<br>(0 to 0)	0.0%<br>(0.0% to 0.0%)	7<br>(6 to 9)	0.0%<br>(0.0% to 0.0%)
**Occupational exposure to cadmium: All cancer outcomes**	0<br>(0 to 0)	0.0%<br>(0.0% to 0.0%)	18<br>(15 to 21)	0.0%<br>(0.0% to 0.0%)
Tracheal, bronchus, and lung cancer	0<br>(0 to 0)	0.0%<br>(0.0% to 0.0%)	18<br>(15 to 21)	0.0%<br>(0.0% to 0.0%)
**Occupational exposure to chromium: All cancer outcomes**	1<br>(1 to 1)	0.0%<br>(0.0% to 0.0%)	38<br>(33 to 42)	0.0%<br>(0.0% to 0.0%)
Tracheal, bronchus, and lung cancer	1<br>(1 to 1)	0.0%<br>(0.0% to 0.0%)	38<br>(33 to 42)	0.1%<br>(0.0% to 0.1%)
**Occupational exposure to diesel engine exhaust: All cancer outcomes**	17<br>(15 to 20)	0.0%<br>(0.0% to 0.0%)	494<br>(434 to 559)	0.0%<br>(0.0% to 0.0%)
Tracheal, bronchus, and lung cancer	17<br>(15 to 20)	0.9%<br>(0.8% to 1.1%)	494<br>(434 to 559)	1.2%<br>(1.1% to 1.4%)
**Occupational exposure to formaldehyde: All cancer outcomes**	1<br>(0 to 1)	0.0%<br>(0.0% to 0.0%)	46<br>(38 to 55)	0.0%<br>(0.0% to 0.0%)
Nasopharynx cancer	0<br>(0 to 0)	0.6%<br>(0.4% to 0.9%)	18<br>(12 to 25)	0.9%<br>(0.6% to 1.2%)
Acute lymphoid leukemia	0<br>(0 to 0)	0.2%<br>(0.2% to 0.3%)	6<br>(4 to 7)	0.2%<br>(0.2% to 0.3%)
Chronic lymphoid leukemia	0	0.1%	1	0.2%

© 2021 Li, N, et al. *JAMA Network Open.*

	(0 to 0)	(0.1% to 0.1%)	(0 to 1)	(0.1% to 0.2%)
Acute myeloid leukemia	0<br>(0 to 0)	0.2%<br>(0.1% to 0.2%)	7<br>(6 to 9)	0.2%<br>(0.2% to 0.3%)
Chronic myeloid leukemia	0<br>(0 to 0)	0.2%<br>(0.2% to 0.2%)	2<br>(1 to 2)	0.3%<br>(0.3% to 0.4%)
Other leukemia	0<br>(0 to 0)	0.2%<br>(0.2% to 0.2%)	11<br>(9 to 13)	0.2%<br>(0.2% to 0.3%)
**Occupational exposure to nickel: All cancer outcomes**	8<br>(1 to 22)	0.0%<br>(0.0% to 0.0%)	238<br>(35 to 607)	0.0%<br>(0.0% to 0.0%)
Tracheal, bronchus, and lung cancer	8<br>(1 to 22)	0.5%<br>(0.1% to 1.2%)	238<br>(35 to 607)	0.6%<br>(0.1% to 1.5%)
**Occupational exposure to PAHs: All cancer outcomes**	4<br>(4 to 5)	0.0%<br>(0.0% to 0.0%)	135<br>(114 to 157)	0.0%<br>(0.0% to 0.0%)
Tracheal, bronchus, and lung cancer	4<br>(4 to 5)	0.3%<br>(0.2% to 0.3%)	135<br>(114 to 157)	0.3%<br>(0.3% to 0.4%)
**Occupational exposure to silica: All cancer outcomes**	48<br>(21 to 76)	2.6%<br>(1.2% to 4.1%)	1,330<br>(595 to 2,080)	3.2%<br>(1.4% to 5.1%)
Tracheal, bronchus, and lung cancer	48<br>(21 to 76)	2.6%<br>(1.2% to 4.1%)	1,330<br>(595 to 2,080)	3.2%<br>(1.4% to 5.1%)
**Occupational exposure to sulfuric acid: All cancer outcomes**	4<br>(1 to 7)	0.0%<br>(0.0% to 0.0%)	124<br>(52 to 225)	0.0%<br>(0.0% to 0.0%)
Larynx cancer	4<br>(1 to 7)	3.2%<br>(1.4% to 5.8%)	124<br>(52 to 225)	3.8%<br>(1.6% to 6.8%)
**Occupational exposure to trichloroethylene: All cancer outcomes**	0<br>(0 to 0)	0.0%<br>(0.0% to 0.1%)	1<br>(0 to 3)	0.0%<br>(0.0% to 0.1%)
Kidney cancer	0<br>(0 to 0)	0.0%<br>(0.0% to 0.1%)	1<br>(0 to 3)	0.1%<br>(0.0% to 0.1%)

Abbreviations: PAF, population attributable fraction; DALY, disability-adjusted life year; PAHs, polycyclic aromatic hydrocarbons.

© 2021 Li, N, et al. *JAMA Network Open.*
